# Multimodal Carbon Monoxide Photorelease from Flavonoids

**DOI:** 10.1021/acs.orglett.3c04141

**Published:** 2024-01-16

**Authors:** Andrea Ramundo, Martina Hurtová, Igor Božek, Zuzana Osifová, Marina Russo, Bokolombe Pitchou Ngoy, Vladimír Křen, Petr Klán

**Affiliations:** † Department of Chemistry, Faculty of Science, Masaryk University, Kamenice 5, 625 00 Brno, Czech Republic; ‡ RECETOX, Faculty of Science, Masaryk University, Kamenice 5, 625 00 Brno, Czech Republic; § Laboratory of Biotransformation, Institute of Microbiology of the Czech Academy of Sciences, Vídeňská 1083, 142 00 Prague, Czech Republic; ∥ Institute of Organic Chemistry and Biochemistry of the Czech Academy of Sciences, Flemingovo nám. 542, 166 00 Prague, Czech Republic

## Abstract

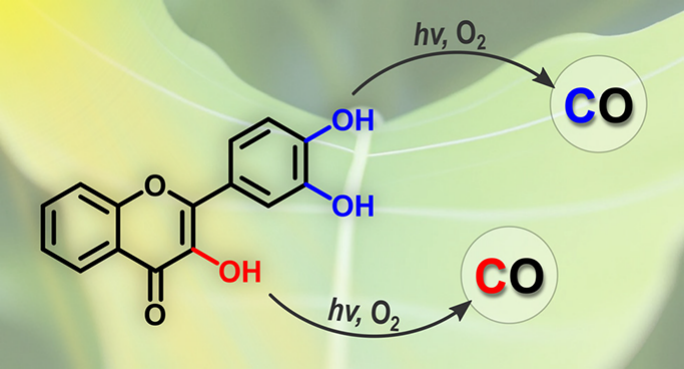

Photooxygenation of flavonoids leads to the release of carbon monoxide (CO). Our structure–photoreactivity study, employing several structurally different flavonoids, including their ^13^C-labeled analogs, revealed that CO can be produced via two completely orthogonal pathways, depending on their hydroxy group substitution pattern and the reaction conditions. While photooxygenation of the enol 3-OH group has previously been established as the CO liberation channel, we show that the catechol-type hydroxy groups of ring B can predominantly participate in photodecarbonylation.

Flavonoids are polyphenolic secondary metabolites found essentially in all plant tissues. Due to their antioxidant, anti-inflammatory, antimutagenic, and anticarcinogenic properties and their generally no or low toxicity, they are valuable in many biotechnological, pharmaceutical, or medical applications.^1^ Their general structure consists of two phenyl rings (A and B) and one heterocyclic ring (C) bearing H, OH, or OCH_3_ substituents in all available positions (Figure 1).

**Figure 1:**
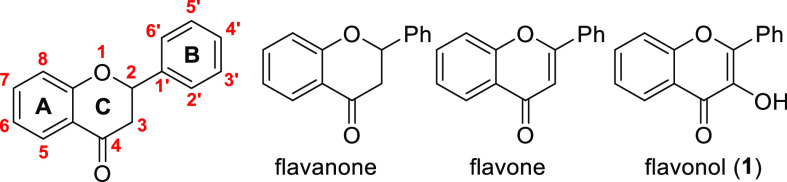
Flavonoid structures discussed in this work.

Flavonoids are natural photoprotectants^2^ and scavengers of radicals and reactive oxygen species,^3^ and their excited states offer rich photochemistry thanks to the diversity of functional groups.^4^

Oxygenation is a characteristic reaction of flavonol (**1**, 3-hydroxyflavone; Figure 1) derivatives. Quercetin (**2**, 3,3′,4′,5,7-pentahydroxyflavone) is readily degraded by fungi, accompanied by the formation of carbon monoxide (CO),^5^ and is even slowly oxidized by air O_2_ in a basic aqueous solution in the dark.^6^ It has been shown that the photoinduced oxygenation of flavonols involves several reaction pathways influenced by pH, as they can exist in acid and base forms^7^ (Scheme 1, in blue). Matsuura proposed that photooxygenation of the acid form proceeds via reaction of a triplet excited state, formed by excited-state intramolecular proton transfer (ESIPT)^8^ and intersystem crossing (ISC), with ground-state O_2_ via an endoperoxide intermediate that rearranges to give CO and salicylic acid ester (Scheme 1, *path A*).^9,10^ We have shown that the conjugate base of flavonol derivatives undergoes an analogous oxidative CO release in polar protic solvents (*path B*).^7^ In addition, singlet oxygen (^1^O_2_) produced by triplet sensitization efficiently oxidizes the conjugate base, yielding the same products (*path C*),^7,9,11^ whereas the acid form is essentially unreactive.^7^

**Scheme 1:**
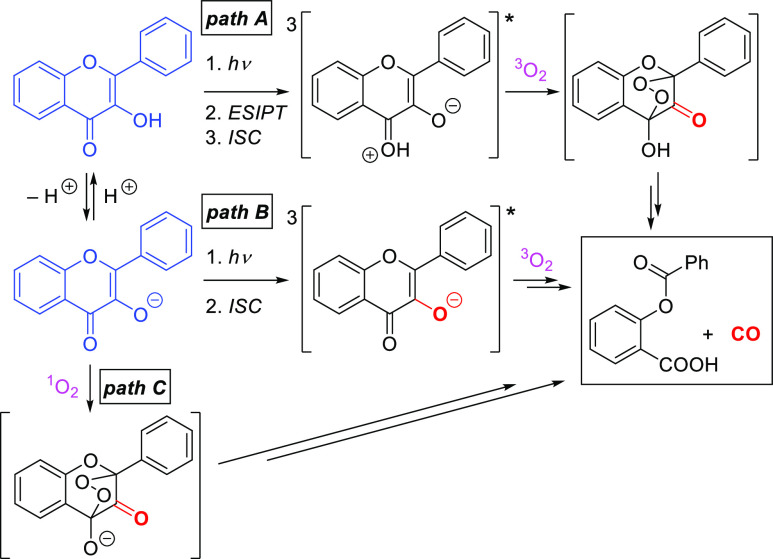
Photoinduced Oxygenation of Flavonol

CO, formed endogenously by oxidative heme degradation, is one of the essential cell signaling molecules that participates in various physiological processes in mammals.^12^ CO is also produced in plants during photorespiratory metabolism^13^ and shows signaling effects by increasing plant resistance to abiotic stress.^14^ Given the widespread occurrence of flavonoids in the plant world and their putative potential to release CO (as photoactivatable CO-releasing molecules, photoCORMs^15^), it seems logical to consider the potent and versatile functions of CO-mediated flavonoids in plant biology and medicine.

The polyphenolic complex structures of natural flavonoids carry several hydroxy groups in all A, B, and C rings. Because mechanistic studies have so far only been performed on simple flavonol structures, we decided to thoroughly study the photooxygenation of several naturally occurring as well as synthetic flavone derivatives to find out how individual functional groups influence their reactivity. The chosen methods included a detailed study of their spectroscopic and photochemical behavior using steady-state and time-resolved methods as well as tracking the photorelease of CO from isotopically labeled derivatives.

Five structurally distinct natural flavonoids, quercetin (**2**), 3′,4′-dihydroxyflavonol (**3**), galangin (**4**), luteolin (**5**), and taxifolin (**6**) (Figure 2), share a typical flavonoid skeleton but differ in the 3,3′,4′,5,7-hydroxy group pattern and the C-2–C-3 bond order. These structural differences have been reported to have significant implications for their physicochemical and biological properties,^16^ such as antioxidant activity.^4^ Flavonols **1** and **7** and its thione analog **8**(17) bear only one C-3 hydroxy group. The 3′-OH and 4′-OH groups in **9** are protected as methoxy groups.

**Figure 2:**
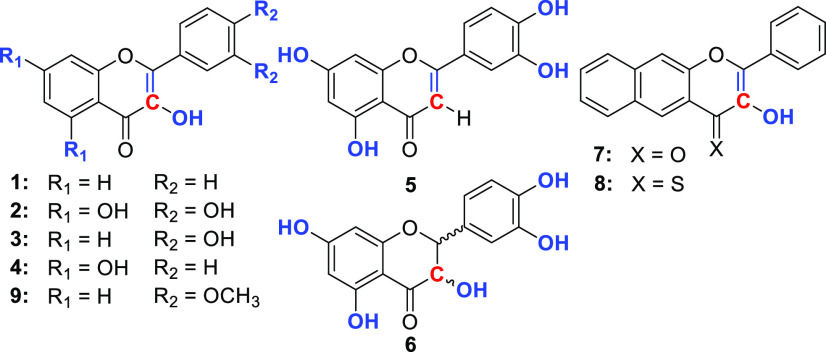
Flavonoids **1**–**9**. The 3-^13^C positions in the isotopically labeled derivatives are marked in red.

Thanks to several hydroxy groups, the acid–base properties of flavones **2**, **3**, **4**, **5**, and **6** are very complex. For example, the OH groups of ring A of quercetin are more acidic (p*K*_a_ ≈ 6–7) than those of ring B (p*K*_a_ ≈ 9).^18,19^ The acidity of the 3-hydroxy groups depends strongly on the overall molecular structure, with p*K*_a_ values ranging from 8.7 for the parent flavonol (**1**)^18^ to more than 13 for quercetin (**2**).^19^ The UVA absorption of the undissociated flavonols (acid forms) **2A**, **3A**, and **4A** (λ_max_ = 355–370 nm) and luteolin (**5A**) (344 nm) is absent in taxifolin, which lacks the C-2–C-3-double bond (**6A**) (290 nm). The π-extended flavonol **7A** and thione analog **8A** have bathochromically shifted absorption (401 and 477 nm, respectively).

The OH groups of the studied compounds are not dissociated in pure methanol (Figures S27–S35). Upon the addition of 6 equiv of NaOCH_3_ as a base, the absorption band maxima were bathochromically shifted (to give the corresponding base forms **2B**, **3B**, **4B** (∼27–51 nm), **5B** (55 nm), **6B** (37 nm); Table 1 and Figures S27–S35), attributed to the deprotonation of at least one OH group. A mixture of the neutral and monoanionic forms was observed for **2** in PBS (5% DMSO, pH 7.4; Figure S36).

**Table 1:** CO Photorelease from Flavonoids

		CO yield/equiv ([^12^CO]:[^13^CO])^c^	
compound (solvent)^a^	λ_abs_/nm (ε /10^4^)^b^	dir	sens
**1A**	344 (1.7)	0.05 (0:100)	0.62 (0:100)
**1B**	406 (1.5)	0.03 (0:100)	0.63 (0:100)
**2A**	370 (2.2)	0.28 (7:93)	0.15 (87:13)
**2B**	397 (1.9)	0.05 (80:20)	0.20 (90:10)
**2** (PBS)^d^	378 (1.1)	0.11^e^ (82:18)	0.70^e^ (95:5)
**2** (PBS)^d^	n.a.	0.23^e^^,^^f^ (3:97)	n.a.
**3A**	366 (2.2)	0.27 (56:44)	0.20 (30:70)
**3B**	417 (1.1)	0.14 (79:21)	0.56 (63:37)
**4A**	355 (1.4)	0.24	0.30
**4B**	384 (1.2)	0.12	0.65
**5A**	344 (1.9)	0	0.40 (100:0)
**5B**	399 (1.4)	0.34 (97:3)	0.99 (94:6)
**6A**	290 (2.2)	n.a.	0.35 (100:0)
**6B**	327 (2.4)	0.20 (95:5)	0.80 (96:4)
**7A**	401 (1.0)	0.80 (0:100)	0.68 (0:100)
**7B**	472 (1.1)	0.55 (0:100)	0.65 (0:100)
**8A**	477 (1.8)	0.98 (3:97)	0^g^
**8B**	544 (1.2)	0.05 (0:100)	0.20 (0:100)^g^
**9A**	361 (2.1)	0.10 (0:100)	0.40 (0:100)

a Methanol (**1A**–**9A**) or basic methanol (**1B**–**8B**; 6 equiv NaOCH_3_).

b Absorption maxima λ_abs_/nm and molar absorption coefficients ε/10^4^ L mol^–1^ cm^–1^.

c Chemical yields of released CO in equiv upon direct irradiation (dir) (λ_irr_ = 365–535 nm irradiated to the tails of the abs. maxima) or photosensitization (sens) (rose bengal, 5 μM; λ_irr_ = 535 nm) to complete conversion. The concentration ratios [^12^CO]:[^13^CO] released from 3′-^13^C-labeled derivatives are in parentheses.

d Compound **2** in PBS (5% DMSO, pH 7.4, 10 mM, *I* = 100 mM) exists as a mixture of **2A** and **2B** (λ_irr_ = 395 nm).

e Corrected for dark CO production.

f DABCO as a ^1^O_2_ quencher (10 mM) added. n.a. = not measured.

g Methylene blue (5 μM) sensitization.

When irradiated directly (dir) in methanol at 395 nm, undissociated flavonols (acid forms) **2A**, **3A**, and **4A** produced CO with similar chemical yields of 0.24–0.28 equiv (Table 1). CO release from **3A** and **4A** was more efficient (the quantum yields of CO production (Φ_CO_) were 0.0013 and 0.0018, respectively) than from **2A** (0.0003) but much less efficient than from **7A** (0.03).^7^ Such low quantum efficiencies are most probably connected to ESIPT,^8^ responsible for the ultrafast nonradiation decay demonstrated for quercetin.^20,21^ Luteolin (**5**) was photostable under the same conditions, and taxifolin (**6**) had no absorption above 350 nm; thus, we did not study its photochemistry. Parent flavonol **1A** released only 0.05 equiv of CO, while its naphthyl derivative **7A** gave a larger chemical yield (0.80 equiv) and exhibited a higher efficiency (Φ_CO_ = 0.03).^7^ We inspected the cause of this nonproductive photodegradation and found that an adduct of the nucleophilic attack of methanol on the C-2 carbon (ring C) of **2A** was formed (Figure S44). On the other hand, thione **8A** showed nearly quantitative CO production (0.98 equiv) with an exceptionally high quantum efficiency of 0.43.^17^ This excellent result thus reflects the compound’s ability to suppress unwanted side processes, as also primarily observed for the π-extended flavonol **7**,^7^ and possibly enhanced intersystem crossing due to the heavy-atom effect of the sulfur atom.

The photochemical activities (including CO production) of flavonols **1**(22) and **7**(23) and flavone (**5**)^24,25^ have been associated with their triplet excited states. We used nanosecond transient absorption spectroscopy to determine the triplet lifetimes of compounds **2A**, **3A**, and **5A** in degassed methanol. Compounds **2A** and **5A** have relatively short lifetimes (140 and 910 ns, respectively), whereas **3A** without OH groups on ring A decayed remarkably slowly (77 μs) (Figures S45–S50). An efficient nonradiative deactivation pathway of the ESIPT state, as reported for **1**,^26^ and a solvent-mediated hydrogen-transfer deactivation thanks to the increased number of OH functionalities seem to be the most reasonable explanations for such short lifetimes.

Some triplet-excited flavonols in protic solvents were reported to sensitize singlet oxygen,^7,23^ whereas their ground states are known to react with ^1^O_2_.^7,10,11^ The quantum yield of ^1^O_2_ production (Φ_Δ_) from triplet excited **2A** in methanol was found to be very small (∼10^–4^), indicating an inefficient process 3 orders of magnitude lower than Φ_Δ_ found for **7A** (0.14^23^). Nevertheless, we investigated CO release in the reaction of selected flavonoids with ^1^O_2_ produced by an external ^1^O_2_ sensitizer (rose bengal; sens; Table 1). While both **1A** and **7A** in methanol reacted with ^1^O_2_ with a higher CO yield of ∼0.65 equiv, the yields from **2A**, **3A**, and **4A** were relatively moderate (0.15–0.30 equiv). Surprisingly, thione **8A** was unreactive under the same conditions and was not investigated further.

Both **5A** and **6A** released even more CO upon sensitization (0.40 and 0.35 equiv, respectively). They lack an enol hydroxy group (3-OH, ring C) and yet photorelease CO, suggesting that different structural features were involved in photooxygenation. This partly contradicts the reported study on the efficiency of singlet oxygen quenching of selected flavonoids, which showed that the ^1^O_2_ physical quenching efficiency by ground-state flavonoids is mainly controlled by the presence of a catechol group (ring B), while the OH group on ring C is predominantly responsible for their chemical reactivity.^27^

CO was also photoproduced from flavonoids with an excess of a base that dissociated the most acidic OH group(s) (NaOCH_3_, 6 equiv; Table 1). In general, the base forms of flavonols gave lower CO yields, which must be related to the alternative photodegradation pathways discussed above. However, much higher CO yields were obtained in the presence of ^1^O_2_ in PBS (pH 7.4, almost 1 equiv; Table 1).

In addition, we investigated the reaction kinetics of **2A** in methanol with ^1^O_2_ (*k*_Σ_), and with a rate constant of *k*_Σ_ ∼ 10^6^ M^–1^ s^–1^ and an estimated quantum yield of photodecarbonylation by self-sensitization of ∼10^–6^ for **2A**, CO production via ^1^O_2_ oxygenation is 300 times less efficient than the reaction of the triplet state with ^3^O_2_ (Scheme 1, *path A*).

To fully understand the CO release mechanism and identify the corresponding carbon atom source, a series of isotopically labeled flavone derivatives featuring ^13^C at the C-3 position were synthesized (>99% enrichment; Figure 2 and Scheme S1). The ^13^C-labeled starting material for the synthesis of compounds ^13^**2**, ^13^**5**, and ^13^**6** (the index denotes the labeled compound) was prepared by Friedel–Crafts acetylation of trimethoxybenzene with acetyl-2-^13^C-chloride.^28^^13^C-labeled luteolin (^13^**5**) was synthesized using a modified reported method,^29^ which involved Claisen–Schmidt condensation of 1-(2,4,6-trimethoxyphenyl)ethan-1-one-2-^13^C and 3,4-dimethoxybenzaldehyde and the subsequent cyclization of a chalcone product (Scheme S3). Taxifolin (^13^**6**) was prepared from 1-(2,4,6-tris(methoxymethoxy)phenyl)ethan-1-one-2-^13^C as a starting material for Claisen–Schmidt condensation and subsequent peroxidation of the resulting chalcone and its cyclization (Scheme S4).^30^ The C-2–C-3 bond of ^13^**6** was oxidized with I_2_ in AcOH/AcOK to give quercetin (^13^**2**), employing an analogous method used for the oxidation of silybin.^31^ Compounds **3** and ^13^**3** were prepared using Claisen–Schmidt condensation of 2′-hydroxyacetophenone-2-^13^C, synthesized by acetylation of phenol with 2-^13^C-acetyl chloride, followed by Fries rearrangement and oxidative cyclization (Schemes S2 and S6). Flavonols ^13^**1** and ^13^**9** were prepared using Claisen–Schmidt condensation followed by cyclization with H_2_O_2_ (Schemes S5 and S8).^23^ 1-(3-Hydroxynaphthalen-2-yl)ethan-1-one-2-^13^C, as a synthetic intermediate, was obtained by the reaction of *in situ*-generated ^13^CH_3_Li with 3-hydroxy-2-naphthoic acid. The Claisen–Schmidt condensation with benzaldehyde gave 3-hydroxy-2-phenyl-4*H*-benzo[*g*]chromen-4-one-3-^13^C (^13^**7**). The thione group at C-4 (^13^**8**) was introduced using Lawesson’s reagent (Scheme S7).^17^

The concentration ratios of released ^12^CO/^13^CO were quantified by headspace GC-MS. A first look at the data in Table 1 suggested that flavonols bearing the OH group only at ^13^C-3 released isotopically pure or almost pure ^13^CO under all circumstances, including photosensitization. This confirms the proposed mechanism^7^ of CO release from flavonols (**1**, **7**, **8**), which occurs exclusively via oxygenation of ring C (Scheme 1, *pathways A–C*).

Concomitant release of ^12^CO and ^13^CO was observed from ^13^**2** and ^13^**3** (Table 1), supporting the involvement of a new mechanistic pathway suggested by photolysis of **5** and **6** that does not bear the enol 3-OH group. Indeed, ^13^**5** and ^13^**6** were almost exclusive producers of ^12^CO.

The ^12^CO/^13^CO ratios were markedly influenced by both the flavonoid structure and the reaction conditions. The specific behavior was very pronounced for quercetin (^13^**2**), which produced predominantly ^13^CO when directly irradiated in methanol, whereas ^12^CO was the major product obtained upon sensitization, especially in PBS (pH 7.4), where both acid and base forms exist in a ratio of about 1:1^19^ (Figure S36). (Note: CO was detected in small amounts (0.06) during the same period of time in the dark, as also reported for moderately basic media before;^6^ therefore, the photodecarbonylation yield shown in Table 1 is corrected.) When **2** in PBS was irradiated in the presence of a large excess of a ^1^O_2_ trap (DABCO, 10 mM), essentially only ^13^CO was released. This means that different rings/sites of the molecules were swapped as the CO source by reaction conditions, although irradiation always leads through a common intermediate, the excited triplet state. In addition, CO was not liberated in the presence of ascorbic acid as an unselective trap of reactive oxygen species (ROS) and oxidation intermediates, which most probably include peroxo compounds (e.g., Scheme 1).

In contrast to ^13^**3**, which generates both ^12^CO and ^13^CO, ^13^**9** with the protected 3′,4′-hydroxy groups produced isotopically pure ^13^CO under all reaction conditions. The hydroxy groups on ring B in ^13^**3** must thus be responsible for the release of ^12^CO. This is also valid for all remaining flavonoids ^13^**2**, ^13^**5**, and ^13^**6** with the 3′,4′-hydroxy-substituted ring B. Another important fact that emerged from the measured data is the maximum yield of CO, which never exceeded 1 equiv. Therefore, we examined the reactivity of catechol-containing model compounds toward oxygenation. Substituted catechols are known to react with ^1^O_2_ via a type II photooxygenation, possibly via exoperoxide intermediates, which rearrange to *o*-quinone derivatives and other oxidation products (Scheme 2).^32,33^ In addition, *o*-quinones were reported to undergo photodecarbonylation by visible-light irradiation,^34^ and CO was shown to be generated from humic acid-containing catechol under irradiation.^35^ To prove that the catechol group releases CO upon ^1^O_2_ sensitization, photooxygenation of 1,2-dihydroxybenzene (catechol) with rose bengal as a sensitizer was carried out under different conditions (see the Supporting Information). The CO yield was found to be ∼0.1 equiv in methanol and increased to 0.38 equiv in the presence of a base (NaOCH_3_, 6 equiv; no CO is liberated in the dark). The yield obtained in PBS was ∼0.3 equiv.

**Scheme 2:**
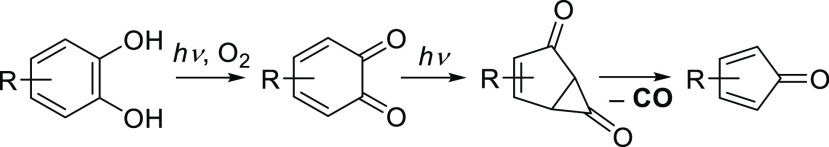
Possible Release of CO from Catechol via Photooxygenation^33^ and Photodecarbonylation^34^

In conclusion, this study changes our view of the photooxygenation of flavonoids that leads to the release of carbon monoxide. We found that the previously established mechanism involving the enol 3-OH group of ring C can be accompanied or even replaced by photodecarbonylation involving the catechol group of ring B. The extent of these orthogonal photooxygenation pathways depends on the pH, solvent, and photoinitiation type. Knowledge of the photooxygenation mechanism is of paramount importance when considering the application of flavonoids as photoCORMs, and it may help to elucidate the mechanisms of release of CO from flavonoids in living plants.

## Data Availability

The data underlying this study are available in the published article and its Supporting Information.

## Supporting Information Available

Materials and methods; synthesis of flavonoids; NMR, optical, transient, and HRMS spectra (PDF)
