# Supplementary material for: Multimodal Carbon Monoxide Photorelease from Flavonoids
Source: Org Lett. 2024 Jan 16;26(3):708–12. doi: 10.1021/acs.orglett.3c04141 (PMC10825817; doi:10.1021/acs.orglett.3c04141)
Supplement: Supplementary file 1 — ol3c04141_si_001.pdf [file ol3c04141_si_001.pdf]

Supporting Information

Multimodal Carbon Monoxide Photorelease from Flavonoids

Andrea Ramundo,[†,‡] Martina Hurtová,[¶] Igor Božek,[†,‡] Zuzana Osifová,[§] Marina Russo,[†,‡] Bokolombe Pitchou Ngoy,[†,‡] Vladimír Křen,[¶,*] and Petr Klán[†,‡,*]

[†] Department of Chemistry, Faculty of Science, Masaryk University, Kamenice 5, 62500, Brno, Czech Republic.

[‡] RECETOX, Faculty of Science, Masaryk University, Kamenice 5, 62500, Brno, Czech Republic.

[¶] Institute of Microbiology of the Czech Academy of Sciences, Laboratory of Biotransformation, Vídeňská 1083, 142 00 Prague, Czech Republic.

[§] Institute of Organic Chemistry and Biochemistry of the Czech Academy of Sciences, Flemingovo nám. 542, 166 00 Prague, Czech Republic.

Corresponding authors: * Vladimír Křen (kren@biomed.cas.cz); Petr Klán (klan@sci.muni.cz)

Contents

1. Methods S3
2. Synthesis of Flavonoids S6
3. NMR Spectra S17
4. HPLC Analyses S26
5. HR-MS S30
6. UV-Vis Absorption Spectra S33
7. Photoproduct Analyses S38
8. Transient Absorption Spectroscopy S39
9. Emission Spectra of the Irradiation Sources S42
10. GC-Headspace Chromatograms S43
11. References S44

1. Methods

Materials and General Methods. Procedures involving oxygen- or moisture-sensitive materials were performed with anhydrous solvents (*vide infra*) under an argon atmosphere in flame-dried flasks using a Schlenk standard technique. The reaction mixtures were heated using Al heat blocks. Analytical TLC was performed on Al plates (Silica Gel 60 F_{254}). Natural flavonoids **2**, **4**, **5**, and **6** were purchased with declared purity above 98%. The commercially available starting materials phenol (97%), 1,3,5-trimethoxybenzene (99%), acetyl-2-^{13}C-chloride (99 atom % ^{13}C), and 3-hydroxy-2-naphthoic acid (98%) were purchased from Sigma Aldrich (Darmstadt, Germany) and used without further purification. Anhydrous solvents were purchased from Acros Organics (Morris Plains, NJ, USA). Analytical HPLC analyses of final compounds were carried out using the Shimadzu Prominence System (Shimadzu, Kyoto, Japan). An ARION Polar C18 HPLC column (3 μm, 100 × 3 mm i.d.; Chromservis, Prague, Czech Republic) coupled with an ARION guard column (5 × 4 mm, Chromservis, Prague, Czech Republic) was used. Mobile phase A $CH_3CN/H_2O/HCOOH$ (5:95:0.1), and phase B $CH_3CN/HCOOH$ (100:0.1) were employed in the analyses unless otherwise stated. Preparative HPLC separations were performed using a polymer-based SEC column at a 5 mL/min methanol flow (isocratic). NMR spectra were recorded on a Bruker Avance III^{TM} HD 500 MHz spectrometer equipped with a cryoprobe (^{1}H 500, ^{13}C 125.7) and the chemical shifts values were referenced to DMSO-d_6 signal (2.50 ppm for ^{1}H and 39.52 ppm for ^{13}C) at 25 °C. The signal in DMSO-d_6 was used as a reference (δ_H = 2.499 ppm, δ_C = 39.46 ppm). The exact masses were obtained using an accurate-mass TOF LC-MS (electrospray or atmospheric pressure chemical ionization) in a positive or negative mode. An HPLC system with a C18-RP column (150 × 4.6 mm, 3.5 μm) was employed for HPLC-UV/vis analyses. The mobile phase consisted of water (0.1% TFA) and CH_3CN or methanol, and gradient elution was performed at a flow rate of 1.000 mL min^{-1}. UV-vis spectra were obtained using a UV-Vis spectrometer with 1.0 cm quartz cuvettes. Photon fluxes were measured using a calibrated Si-photodiode. Reactions under microwave irradiation were performed in the Biotage Initiator + microwave reactor (Biotage, Uppsala, Sweden) using Biotage microwave reaction vessels (borosilicate glass, 0.2–20 mL) sealed with aluminum cap and Teflon septa. The reaction mixture temperature was monitored automatically by a microwave reactor.

General Procedure for Irradiation in UV Cuvettes. A sample was dissolved in the specified solvent (3 mL) and placed in a 1.0 cm quartz PTFE screw-cap cuvette containing a stirring rod. The solution was continuously stirred while irradiated by a custom-made LED array at the indicated wavelength, placed at the 2.5 cm distance from the reaction vessel. The progress of photoreactions was monitored at specific time intervals using UV-vis spectroscopy with a diode-array spectrophotometer.

Determination of CO Yields Upon Direct Irradiation. The sample solution in the given solvent (800 μL) was placed in sealed GC vials fitted with PTFE septa. The vials were then irradiated with an LED array over the given periods and the conversion was monitored by RP-HPLC. The amount of CO released was measured using a GC-headspace instrument equipped with a TIC/MS detector operating in SIM mode and a GC column (5Å, 30 m length, 0.53 mm inner diameter, and 50 μm df). The instrument was calibrated using the quantitative photoreaction of cyclopropenone photoCORM[1] (20–400 μL, concentration ~50 μM in methanol). The CO yields were determined as the total chemical yields and expressed as

equivalents of CO released from 1 equivalent of the reactant. Two independent measurements were done for each sample, and the results were averaged. The standard error in all cases was found to be below 10%. The $^{12}CO/^{13}CO$ concentration ratios from the isotopically labeled compounds were determined via the independent quantification of ^{12}CO (at 28 *m/z*) and ^{13}CO (at 29 *m/z*) signals.

Determination of CO Yields Upon Sensitization. A solution containing the sample (100 μM; flavonoid or catechol) and rose bengal (RB; 5 μM) as a singlet-oxygen sensitizer in the given solvent was placed in sealed GC vials fitted with PTFE septa. The vials were irradiated with an LED array at 535 nm over the given time period, and the conversions were monitored by RP-HPLC. The amount of CO released was measured using a GC-headspace using the same method described above.

Quantum Yield of CO Release (Φ_{CO}). A solution of the sample in methanol (c = 5 μM) in a closed GC vial fitted with a PTFE septum was irradiated using a xenon lamp through a monochromator set to the corresponding wavelength. The light beam (~7 mm^2) was collimated through the bottom of the vial onto a calibrated Si-photodiode. The total irradiation time was chosen to reach a <10% conversion. The photorelease quantum yield (Φ_{CO}) was obtained as an absolute number from the amount of CO released against the number of photons absorbed by the sample in the given time. The total amount of photons absorbed by the sample $n_{\mathrm{p}}^{\mathrm{abs}}$ was determined according to the following formula:

$$n_{\mathrm{p}}^{\mathrm{abs}} = t q_0 \int_0^{\infty} (1 - 10^{-A(\lambda)})\, I_{\mathrm{norm}}^{\mathrm{em}}(\lambda) d\lambda$$

where t is the time, $A(\lambda)$ is the sample absorbance at the wavelength λ, $I_{\mathrm{norm}}^{\mathrm{em}}(\lambda)$ is the emission spectrum of the light source at the wavelength λ normalized to a unit area (m^2), and q_0 is the absolute photon flux calculated by:

$$q_0 = \frac{I}{N_{\mathrm{A}} hc} \int_0^{\infty} \frac{\lambda I_{\mathrm{norm}}^{\mathrm{em}}(\lambda)}{R(\lambda)} d\lambda$$

where I (in A) is the current measured by a calibrated Si-photodiode when the light beam goes through a blank solution of the solvent, N_A is the Avogadro number, h is the Planck constant, c is the speed of light, λ is the wavelength, $I_{\mathrm{norm}}^{\mathrm{em}}(\lambda)$ is the emission spectrum of the light source at the wavelength λ normalized to a unit area (m^2), and R is a wavelength-dependent instrument responsivity (in A W^{-1}). The amount of CO released was determined by headspace GC-MS. Three independent measurements were performed for each sample.

Quantum Yield of $^1O_2$ Production (Φ_Δ). A solution of furfuryl alcohol (FFA; 1 mM) and a flavonoid derivative (100 μM) or tetra(4-sulfonatophenyl)porphyrin (TPPS; 40 μM) in methanol was prepared. The solution was stirred and exposed to LED irradiation at 395 nm. The consumption of FFA by singlet oxygen was determined by HPLC analysis using a reported method.[2] The quantum yield of singlet oxygen production (Φ_Δ) was calculated from the quantum yield of TPPS as a reference ($\Phi_\Delta = 0.69$)[3] and the bimolecular rate constant (k_r) of the reaction between FFA and $^1O_2$ ($k_r = 1.03 \times 10^8$ M^{-1} s^{-1}).[4]

Reactions with Singlet Oxygen (k_Δ). A methanol solution containing the sample (40–100 μM; A~1 at λ_{max}) and rose bengal (RB; 5 μM) as a singlet-oxygen sensitizer was transferred to a 3.5 mL quartz cell with a 1.0 cm path length. The solution was irradiated with an LED array at 535 nm, and UV-vis absorption spectra were recorded at specified time intervals. Methanol solutions containing DPBF (45 μM) and RB (5 μM) were prepared in the same way and irradiated under the same conditions, and UV-vis absorption spectra were recorded at specified time intervals. The rate constants of DPBF and flavone decomposition were determined from the decay of the corresponding absorption maxima (λ_{max}). The bimolecular reaction rates of flavone with singlet oxygen ($^1O_2$) were calculated using the known rate constant of singlet oxygen quenching in methanol ($k_d = 9 \times 10^4$ s^{-1}) and the bimolecular reaction rate constant of DPBF with singlet oxygen ($k_r = 1.2 \times 10^9$ M^{-1} s^{-1}).[4]

Transient Absorption Spectroscopy. The nanosecond laser flash photolysis setup was employed in a 90° configuration for the pump and probe beams. Laser pulses with a duration of ≤700 ps were generated at 355 nm (160 mJ) from an Nd:YAG laser. The laser beam was dispersed into a modified fluorescence cuvette (40 × 10 mm), positioned horizontally. An over-pulsed Xe arc lamp served as a probe light source. Kinetic decay traces were recorded using a photomultiplier, whereas transient absorption spectra were obtained using an ICCD camera equipped with a spectrograph. Prior to the measurements, the samples were degassed by subjecting them to three freeze-pump-thaw cycles. Experiments were conducted at room temperature (20 ± 2 °C) using methanol or aqueous phosphate-buffered saline (PBS, I = 0.1 M, pH = 7.4) as solvent at excitation wavelengths of 355 nm.

Calculation of Quantum Yield of the Reaction with $^1O_2$. The reaction rate between flavonoid **2** and $^1O_2$ can be expressed as:

$$k_r = -\frac{d[\mathbf{2}]}{dt} = \frac{dn(\mathbf{2})}{V\,dt} = k_\Sigma\,[^1O_2]\,[\mathbf{2}] \qquad \text{Eq. S1}$$

where k_r is the rate of decomposition, [**2**] is the concentration of **2**, n(**2**) is the amount of **2** in moles, V is the sample volume, k_Σ is the bimolecular reaction rate of the flavonoid and singlet oxygen, and [$^1O_2$] is the concentration of singlet oxygen.

The quantum yield of **2** photooxygenation is defined by:

$$\Phi_{dec} = \frac{dn(\mathbf{2})}{q^0_{n,p}[1 - 10^{-A(\lambda)}]dt} \qquad \text{Eq. S2}$$

where $q^0_{n,p}$ is the incident photon flux and $A(\lambda)$ is the absorbance of the sample at the given wavelength. Combining Eq. S1 and Eq. S2 gives:

$$\Phi_{dec} = \frac{k_r[^1O_2]\,[\mathbf{2}]\,V}{q^0_{n,p}[1 - 10^{-A(\lambda)}]} \qquad \text{Eq. S3}$$

The production and deactivation of singlet oxygen is given by the following equation:

$$\frac{d[^1O_2]}{dt} = \frac{\Phi_\Delta\, q^0_{n,p}[1 - 10^{-A(\lambda)}]}{V} - k_d\,[^1O_2] - k_\Sigma\,[^1O_2]\,[\mathbf{2}] \qquad \text{Eq. S4}$$

where Φ_Δ is the known quantum yield of singlet oxygen formation from **2** and k_d is the first-order rate constant of quenching by the solvent. Applying steady-state approximation for $[^1O_2]$, we get:

$$[^1O_2] = \frac{q^0_{n,p}\left[1 - 10^{-A(\lambda)}\right]}{V} \times \frac{\Phi_\Delta}{(k_d + k_\Sigma[\mathbf{2}])} \qquad \text{Eq. S5}$$

Finally, inserting Eq. S5 to Eq. S3 gives:

$$\Phi_{dec} = \frac{k_\Sigma\,[\mathbf{2}]\,\Phi_\Delta}{k_d + k_\Sigma[\mathbf{2}]} \qquad \text{Eq. S6}$$

Employing the values used in the experiments ([**2**] = 100 μM; $k_d = 9 \times 10^4\ s^{-1}$), the calculated value of $\Phi_{dec} = 1.0 \times 10^{-6}$ is obtained for **2**. Hence, the ratio of Φ_{CO} and Φ_{dec} is calculated.

$$\frac{\Phi_{CO}}{\Phi_{dec}} = \frac{3.0 \times 10^{-4}}{1.0 \times 10^{-6}} = 300 \qquad \text{Eq. S7}$$

2. Synthesis of Flavonoids

Scheme S1. General Scheme for the Preparation of Flavonoids

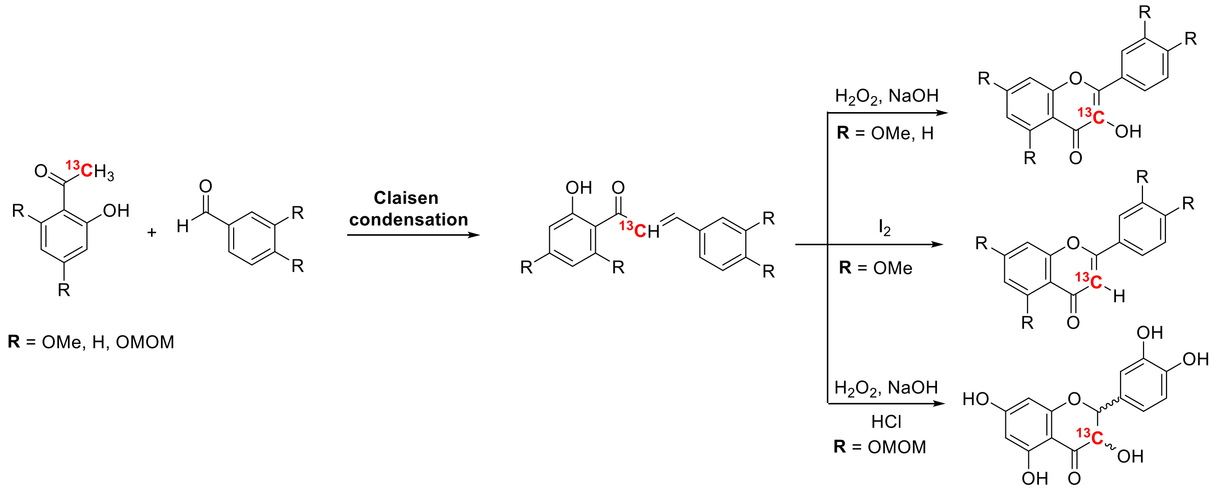

Preparation of 3,3',4'-Trihydroxyflavone (3)

Scheme S2. Preparation of 3.

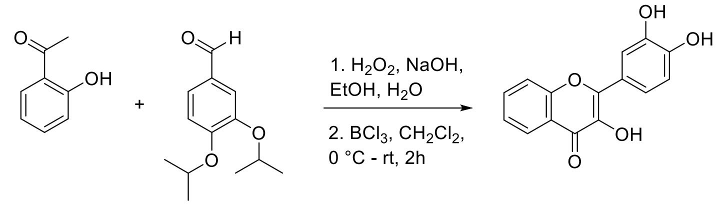

S1 3

3,4-Diisopropoxybenzaldehyde (S1)

3,4-Dihydroxybenzaldehyde (1.0 g, 7 mmol, 1 equiv) and K_2CO_3 (3.0 g, 21 mmol, 3 equiv) were dissolved in DMF (20 mL). 2-Iodopropane (2.2 mL, 21 mmol, 3 equiv) was added. The reaction mixture was stirred at 60 °C for 16 h. The reaction mixture was poured into water and extracted with EtOAc (3 × 100 mL). The organic phase was washed with water (3 × 100 mL), brine (3 × 100 mL), dried over Na_2SO_4, and evaporated *in vacuo*. The residue was purified by FCC (3:1 cyclohexane/EtOAc) to give compound **S1** as a colorless oil (1.4 g, 90%). ^{1}H NMR (500 MHz, DMSO-d_6) δ 9.81 (s, 1H), 7.52 (dd, J = 8.4, 2.0 Hz, 1H), 7.40 (d, J = 1.8 Hz, 1H), 7.18 (d, J = 8.5 Hz, 1H), 4.72 (sept, J = 6.1 Hz, 1H), 4.55 (sept, J = 6.1 Hz, 1H), 1.30 (d, J = 6.1 Hz, 6H), 1.27 (d, J = 6.1 Hz, 6H) ppm. The NMR data correspond to those in the literature.[5]

2-(3,4-Dihydroxyphenyl)-3-hydroxy-4*H*-chromen-4-one (3)

2′-Hydroxyacetophenone (442 µL, 3.7 mmol, 1 equiv), 3,4-diisopropoxybenzaldehyde (**S1**, 815 mg, 3.7 mmol, 1 equiv), and KOH (617 mg, 11.0 mmol, 3 equiv) were dissolved in 12 mL of EtOH/H_2O mixture (5:1) in a 20 mL microwave vial. The reaction mixture was irradiated in a microwave reactor at 65 °C for 40 min. The reaction mixture was then cooled to room temperature and H_2O_2 (30% aqueous solution, 1.1 mL, 11.0 mmol, 3 equiv) was added. The reaction mixture was irradiated in a microwave reactor at 40 °C for 20 min. The reaction mixture was poured into water and extracted with EtOAc (3 × 50 mL). The organic layer was washed with brine (3 × 50 mL), dried over Na_2SO_4, and evaporated to dryness *in vacuo*. The residue was dissolved in dry CH_2Cl_2 (5 mL) and cooled to –10 °C. A solution of BCl_3 (1 M in CH_2Cl_2, 3.5 mL, 11 mmol, 3 equiv) was added dropwise. The reaction mixture was stirred at –10 °C for 30 min, then heated to 40 °C and stirred for 2 h. The reaction mixture was then cooled to 0 °C, and an excess of methanol was added. The reaction mixture was evaporated, and the residue was purified by preparative HPLC chromatography (5 mL/min, isocratic) to give the product **3** as a yellow solid (300 mg, 30%). ^{1}H NMR (500 MHz, DMSO-d_6) δ 9.58 (s, 1H), 9.30 (bs, 2H), 8.09 (dd, J = 8.0, 1.6 Hz, 1H), 7.81–7.72 (m, 2H), 7.70 (dd, J = 8.6, 1.1 Hz, 1H), 7.61 (dd, J = 8.5, 2.2 Hz, 1H), 7.45 (ddd, J = 8.1, 7.0, 1.1 Hz, 1H), 6.91 (d, J = 8.5 Hz, 1H) ppm. ^{13}C{^{1}H} NMR (126 MHz, DMSO-d_6) δ 172.5, 154.4, 147.6, 146.1, 145.1, 137.9, 133.4, 124.7, 124.4, 122.3, 121.3, 120.0, 118.2, 115.6, 115.3 ppm. The NMR data correspond to those in the literature.[6]

Preparation of ^{13}C-Luteolin (^{13}5)

Scheme S3. Synthesis of ^{13}5

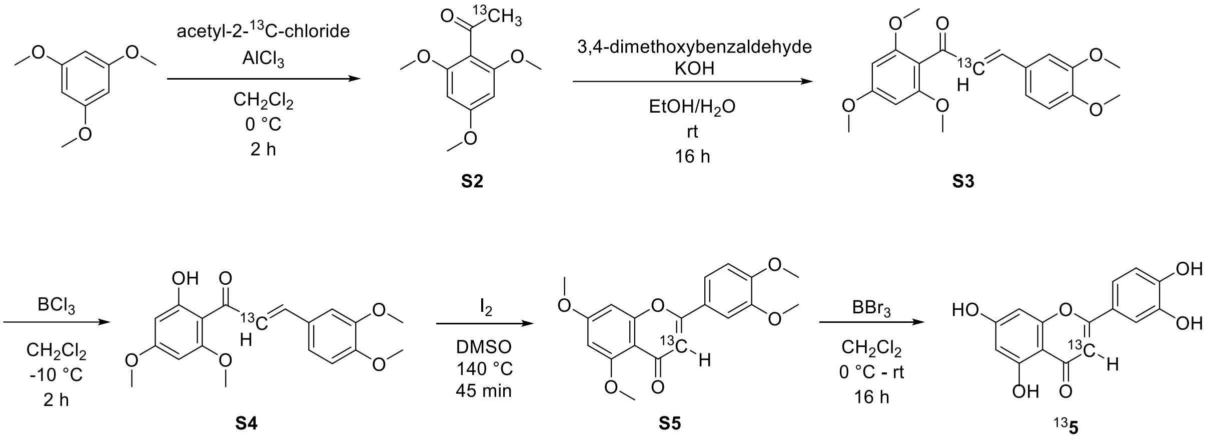

The labeled compounds were prepared according to the previously published method for the preparation of unlabeled luteolin.[7]

1-(2,4,6-Trimethoxyphenyl)ethan-1-one-2-^{13}C (S2)

$AlCl_3$ (555 mg, 4.2 mmol, 1.4 equiv) was added in portions to CH_2Cl_2 (7 mL) at 0 °C. Acetyl-2-^{13}C-chloride (220 µL, 3.1 mmol, 1.05 equiv) was added dropwise. 1,3,5-Trimethoxybenzene (500 mg, 2.9 mmol, 1 equiv) was dissolved in 2 mL of CH_2Cl_2 and added dropwise to the reaction mixture. The reaction mixture was stirred at 0 °C for 2h and then quenched with 10% NaOH until the pH was basic. The solution was filtered through a Celite 545 pad, and the water phase was extracted with CH_2Cl_2 (3 × 100 mL). The organic phase was washed with brine (3 × 100 mL), dried over Na_2SO_4, and evaporated *in vacuo*. The residue was purified by FCC (3:1 cyclohexane/EtOAc) to give compound **S2** as a white solid (400 mg, 64%). ^{1}H NMR (500 MHz, DMSO-d_6) δ 6.25 (s, 2H), 3.79 (s, 3H), 3.73 (s, 6H), 2.30 (d, J = 127.6 Hz, 3H) ppm. The NMR data correspond to the previously reported data for the unlabeled compound.[7]

3-(3,4-Dimethoxyphenyl)-1-(2,4,6-trimethoxyphenyl)-2-prop-2-en-1-one-2-^{13}C (S3)

Compound **S2** (100 mg, 0.5 mmol, 1 equiv) and 3,4-dimethoxybenzaldehyde (87 mg, 0.52 mmol, 1.1 equiv) were dissolved in EtOH (5 mL). The reaction mixture was cooled to 0 °C, and KOH (50% aqueous solution, 10 mL) was added dropwise. The reaction mixture was stirred at room temperature for 16 h. The reaction was cooled to 0 °C, acidified to pH 3 with 4 M HCl (aqueous solution), and extracted with CH_2Cl_2 (3 × 20 mL). The organic phase was washed with water (3 × 20 mL), and brine (3 × 20 mL) and evaporated *in vacuo*. The residue was purified by FCC (1:1 cyclohexane/EtOAc) to give the product **S3** as a white solid (158 mg, 92%). ^{1}H NMR (500 MHz, DMSO-d_6) δ 7.28 (d, J = 2.1 Hz, 1H), 7.17 (dd, J = 8.5, 1.8 Hz, 1H), 7.12 (d, J = 16.2 Hz, 1H), 6.95 (d, J = 8.5 Hz, 1H), 6.87 (d, J = 15.9 Hz, 1H), 6.30 (s, 2H), 3.83 (s, 3H), 3.79 (s, 3H), 3.78 (s, 3H), 3.70 (s, 6H) ppm. The NMR data correspond to the previously reported data for the unlabeled compound.[7]

3-(3,4-Dimethoxyphenyl)-1-(2-hydroxy-4,6-dimethoxyphenyl)-2-prop-2-en-1-one-2-^{13}C (S4)

Chalcone (**S3**, 140 mg, 0.4 mmol, 1 equiv) was dissolved in CH_2Cl_2 (2 mL), and the reaction mixture was cooled to –10 °C. BCl_3 (1 M in CH_2Cl_2, 0.5 mL, 0.5 mmol, 1.1 equiv) was added dropwise, and the reaction mixture was stirred for 1 h at –10 °C. After the starting material was consumed, the reaction mixture was warmed to 0 °C, an excess of methanol was added, and the reaction mixture was stirred for 10 min. 50 mL of water was added to the solution, followed by the addition of $NaHCO_3$ (pH 8). The solution was then extracted with CH_2Cl_2 (3 × 20 mL). The organic layer was washed with water (3 × 20 mL) and brine (3 × 20 mL), dried over anhydrous Na_2SO_4, filtered, and evaporated *in vacuo*. Evaporation of the solvent gave the chalcone **S4** as an orange solid (126.2 mg, 94%). ^{1}H NMR (500 MHz, DMSO-d_6) δ 13.39 (s, 1H), 7.68 – 7.57 (m, 2H), 7.31 – 7.27 (m, 2H), 7.02 (d, J = 8.9 Hz, 1H), 6.18 – 6.10 (m, 2H), 3.88 (s, 3H), 3.83 (s, 3H), 3.82 (s, 3H), 3.81 (s, 3H) ppm. The NMR data correspond to the previously reported data for the unlabeled compound.[7]

2-(3,4-Dimethoxyphenyl)-5,7-dimethoxy-4*H*-chromen-4-one-3-^{13}C (S5)

Chalcone (**S4**, 121 mg, 0.4 mmol, 1 equiv) was dissolved in DMSO (3 mL), and I_2 (8.9 mg, 0.04 mmol, 0.1 equiv) was added. The reaction mixture was heated at 140 °C for 45 min. The reaction mixture was allowed to cool to room temperature, and 40 mL of EtOAc was added. The solution was washed with $Na_2S_2O_3$ (1 M, 3 × 20 mL), followed by brine (3 × 20 mL). The organic phase was dried over anhydrous Na_2SO_4, filtered, and evaporated to dryness *in vacuo*. The crude product was purified by FCC (1:1 toluene/acetone) to give the product **S5** as a white solid (97 mg, 81%).

2-(3,4-Dihydroxyphenyl)-5,7-dihydroxy-4*H*-chromen-4-one-3-^{13}C (^{13}C-luteolin, ^{13}**5**)

Compound **S5** (97 mg, 0.3 mmol, 1 equiv) was dissolved in dry CH_2Cl_2 (5 mL) and cooled to 0 °C. A solution of BBr_3 (1 M in CH_2Cl_2, 2.2 mL, 2.3 mmol, 8 equiv) was added dropwise. The reaction mixture was stirred at rt for 16 h. After completion of the reaction, 10 mL of methanol was carefully added to the flask and left stirring at rt for 10 min. 50 mL of water was added, followed by the addition of $NaHCO_3$ to pH 8. The solution was then extracted with CH_2Cl_2 (3 × 20 mL). The organic layer was washed with water (3 × 20 mL) and brine (3 × 20 mL), dried over anhydrous Na_2SO_4, filtered, and evaporated to dryness *in vacuo*. The residue was purified by preparative HPLC (ASAHIPACK, 5 mL/min, isocratic), affording ^{13}C-luteolin (^{13}**5**) as a yellow solid (30 mg, 40%). ^{1}H NMR (500 MHz, DMSO-d_6) δ 12.98 (s, 1H), 10.83 (s, 1H), 9.92 (s, 1H), 9.40 (s, 1H), 7.43 – 7.40 (m, 1H), 7.40 – 7.38 (m, 1H), 6.89 (d, J = 8.5 Hz, 1H), 6.67 (d, J = 167.8 Hz, 1H), 6.44 (d, J = 2.1 Hz, 1H), 6.19 (d, J = 2.1 Hz, 1H) ppm. ^{13}C{^{1}H} NMR (126 MHz, DMSO-d_6) δ 181.7 (d, J = 58 Hz), 164.1, 163.8 (d, J = 70 Hz), 161.5, 157.3, 149.7, 145.7, 121.5 (d, J = 4 Hz), 119.0 (d, J = 1 Hz), 116.0, 113.4, 106.9, 102.9, 98.8, 93.8 ppm. The NMR data correspond to the previously reported data for the unlabeled compound.[7] HRMS (APCI–) *m/z*: calcd. for $C_{14}{}^{13}CH_9O_6$ $[M-H]^-$ 286.0438, found 286.0440.

Preparation of ^{13}C-Taxifolin (^{13}6) and ^{13}C-Quercetin (^{13}2)

Scheme S4. Synthesis of ^{13}6 and ^{13}2

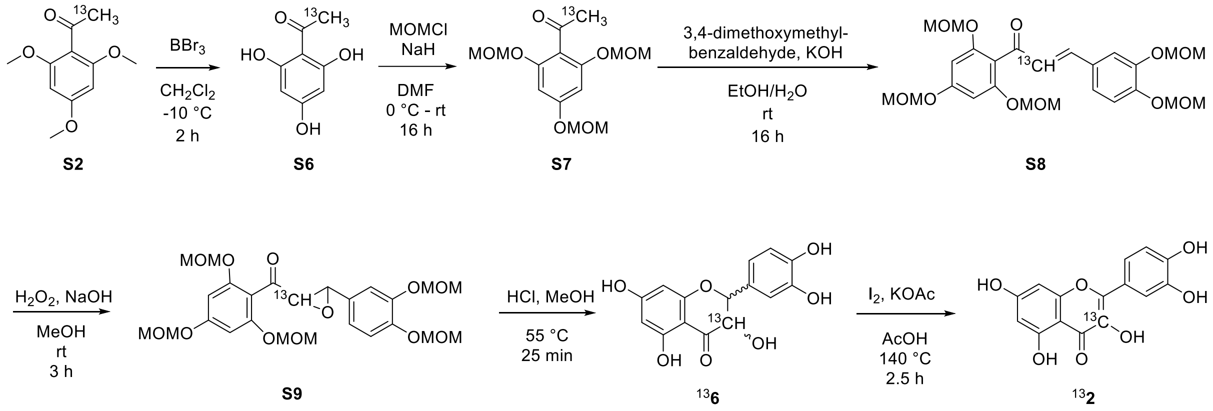

The labeled compounds were prepared according to the previously published method for the preparation of unlabeled taxifolin.[8]

1-(2,4,6-Trihydroxyphenyl)ethan-1-one-2-^{13}C (S6)

Compound **S2** (254 mg, 1.2 mmol, 1 equiv) was dissolved in dry CH_2Cl_2 (4 mL) and cooled to –10 °C. A solution of BBr_3 (1 M in CH_2Cl_2, 4.8 mL, 4.8 mmol, 4 equiv) was added dropwise. The reaction mixture was stirred at –10 °C for 30 min, then heated to 40 °C and stirred for 16 h. The reaction mixture was then cooled to 0 °C, and an excess of methanol was added. The mixture was poured into water followed by the addition of $NaHCO_3$ to pH 8. The solution was then extracted with CH_2Cl_2 (3 × 20 mL). The organic layer was washed with water (3 × 20 mL) and brine (3 × 20 mL), dried over anhydrous Na_2SO_4, filtered, and evaporated to dryness *in vacuo*. The residue was purified by FCC (1:1 cyclohexane/EtOAc) to give the product **S6** as a white solid (155 mg, 75%). ^{1}H NMR (500 MHz, DMSO-d_6) δ 12.22 (bs, 2H), 10.37 (bs, 1H), 5.79 (s, 2H), 2.54 (d, J = 128.9 Hz, 3H) ppm. NMR data correspond with previously reported data for non-labeled compound.[9]

1-(2,4,6-Tris(methoxymethoxy)phenyl)ethan-1-one-2-^{13}C (S7)

NaH (160 mg, 4.0 mmol, 4.5 equiv) was added in portions to a solution of 2,4,6-trihydroxyacetophenone (**S6**, 150 mg, 0.9 mmol, 1 equiv) in dry DMF at 0 °C. Chloromethyl methyl ether (305 µL, 4.0 mmol, 4.5 equiv) was added dropwise. The reaction mixture was stirred at rt for 16 h. The reaction mixture was quenched by adding ice-cold water and extracted with EtOAc (3 × 40 mL). The combined organic layer was washed with distilled water (3 × 40 mL) and brine (3 × 40 mL), and then it was dried over Na_2SO_4, filtered, and evaporated to dryness *in vacuo*. The residue was purified by FCC (3:1 petroleum ether/EtOAc) to give the desired product **S7** as a colorless oil (203 mg, 76%). ^{1}H NMR (500 MHz, DMSO-d_6) δ 6.47 (s, 2H), 5.18 (s, 2H), 5.16 (s, 4H), 3.38 (s, 3H), 3.35 (s, 6H), 2.38 (d, J = 127.9 Hz, 3H) ppm. The NMR data correspond to the previously reported data for the unlabeled compound.[8]

3-(3,4-Bis(methoxymethoxy)phenyl)-1-(2,4,6-tris(methoxymethoxy)phenyl)-2-prop-2-en-1-one-2-^{13}C (S8)

To a solution of benzophenone (**S7**, 200 mg, 0.7 mmol, 1 equiv) in EtOH (3 mL) was added a solution of KOH (114 mg, 2.0 mmol, 3 equiv) in ethanol (2 mL). 3,4-Dimethoxymethylbenzaldehyde (168 mg, 0.7 mmol, 1 equiv) was added to the reaction mixture and the reaction mixture was left to stir at rt for 3 h. Distilled water was added to the reaction mixture and extracted with EtOAc (3 × 20 mL). The combined organic layers were washed with brine (3 × 20 mL), dried over Na_2SO_4, filtered, and evaporated to dryness *in vacuo*. The residue was purified by FCC (1:1 cyclohexane/EtOAc) to give chalcone **S8** as a colorless oil (200 mg, 59%). ^{1}H NMR (500 MHz, DMSO-d_6) δ 7.43 (d, J = 2.1 Hz, 1H), 7.29 (dd, J = 8.5, 2.1 Hz, 1H), 7.18 (d, J = 15.9 Hz, 1H), 7.10 (d, J = 8.5 Hz, 1H), 6.91 (dd, J = 158.0, 16.0 Hz, 1H), 6.52 (s, 2H), 5.24 (s, 4H), 5.21 (s, 2H), 5.13 (s, 4H), 3.41 (s, 3H), 3.39 (s, 3H), 3.39 (s, 3H), 3.27 (s, 6H) ppm. The NMR data correspond to the previously reported data for the unlabeled compound.[10]

(3-(3,4-Bis(methoxymethoxy)phenyl)oxiran-2-yl-2-^{13}C)(2,4,6-tris(methoxymethoxy)phenyl)methanone (S9)

H_2O_2 (30% aqueous solution, 70 µL, 0.9 mmol, 2.5 equiv) and NaOH (2 M aqueous solution, 89 µL, 0.2 mmol, 0.5 equiv) were added to a methanol solution of chalcone (**S8,** 180 mg, 0.4 mmol, 1 equiv), and the reaction mixture was stirred at rt for 3 h. Distilled water was added to the reaction mixture, and it was extracted with EtOAc (3 × 20 mL). The combined organic layers were washed with brine (3 × 20 mL), dried over Na_2SO_4, filtered, and evaporated to dryness *in vacuo*. The resulting epoxide **S9** (177 mg, 95%) was subjected to the next step without further purification.

2-(3,4-Dihydroxyphenyl)-3,5,7-trihydroxychroman-4-one-3-^{13}C (^{13}C-taxifolin, ^{13}**6**)

Epoxide **S9** (170 mg, 0.3 mmol, 1 equiv) was dissolved in 1 M HCl in methanol (10 mL) and stirred at 55 °C for 25 min. Methanol was removed *in vacuo*. Distilled water was added to the residue, and the mixture was extracted with EtOAc (3 × 20 mL). The combined organic layers were dried over Na_2SO_4, filtered, and evaporated to dryness *in vacuo*. The residue was purified by FCC (2:1 toluene/acetone) to give ^{13}C-taxifolin (^{13}**6**) as a white solid (30 mg, 30%). The final product was isolated as a mixture of *trans* and *cis* isomers in the ca 3:1 ratio. ^{1}H NMR (500 MHz, DMSO-d_6) δ 11.90 (bs, 1H), 10.85 (bs, 1H), 9.03 (bs., 1H), 8.97 (bs., 1H), 8.92 (m., 1H), 6.87 (d, J = 1.5 Hz, 1H), 6.76 – 6.68 (m, 2H), 5.91 - 5.88 (m, 1H), 5.85 (d, J = 2.1 Hz, 1H), 5.75 (dd, J = 5.8, 2.4 Hz, 1H), 4.99 – 4.94 (m, 1H), 4.49 (m, 1H), ppm. ^{13}C{^{1}H} NMR (126 MHz, DMSO-d_6) δ 197.2 (d, J = 44 Hz), 166.8, 163.3, 162.5, 145.7, 144.9, 128.0, 119.4, 115.3, 115.1, 100.5 (d, J = 9 Hz), 96.0, 95.0, 83.2, 71.5 ppm. The NMR data correspond to the previously reported data for the unlabeled compound.[10] HRMS (APCI–) *m/z*: calcd. for $C_{14}{}^{13}CH_{11}O_7$ $[M-H]^-$ 304.0544, found 304.0542.

2-(3,4-Dihydroxyphenyl)-3,5,7-trihydroxy-4*H*-chromen-4-one-3-^{13}C (^{13}C-quercetin, ^{13}**2**)

^{13}C-Taxifolin (^{13}**6**, 100 mg, 0.3 mmol, 1 equiv) and KOAc (1.6 g, 15 mmol, 50 equiv) were dissolved in acetic acid (10 mL) and cooled to 0 °C. I_2 (170 mg, 0.6 mmol, 2 equiv) in acetic acid (5 mL) was added dropwise. The resulting mixture was refluxed for 2.5 h. The reaction mixture was poured into ice-cold water, $Na_2S_2O_3$ was added, and it was extracted with EtOAc

(3 × 20 mL). The combined organic layers were washed with water (3 × 20 mL) and brine (3 × 20 mL), dried over Na_2SO_4, and evaporated to dryness *in vacuo*. The residue was purified by preparative HPLC chromatography (ASAHIPACK, 5 mL/min, isocratic) affording ^{13}C-quercetin (^{13}**2**) as a yellow solid (21.4 mg, 22%). ^{1}H NMR (500 MHz, DMSO-d_6) δ 12.49 (s, 1H), 10.78 (bs., 1H), 9.59 (bs, 1H), 9.36 (bs, 1H), 9.30 (bs, 1H), 7.67 (d, J = 2.1 Hz, 1H), 7.53 (dd, J = 8.5, 2.1 Hz, 1H), 6.88 (d, J = 8.2 Hz, 1H), 6.40 (d, J = 1.8 Hz, 1H), 6.18 (d, J = 1.8 Hz, 1H) ppm. ^{13}C{^{1}H} NMR (126 MHz, DMSO-d_6) δ 175.8 (d, J = 58 Hz), 163.9, 160.7 (d, J = 2 Hz), 156.1 (d, J = 3 Hz), 147.7, 146.8 (d, J = 90 Hz), 145.1, 135.7, 121.9 (d, J = 3 Hz), 120.0, 116.0, 115.1, 103.0, (d, J = 11 Hz), 98.2, 93.9 ppm. The NMR data correspond to the previously reported data for the unlabeled compound.[11] HRMS (APCI–) *m/z*: calcd. for $C_{14}{}^{13}CH_9O_7$ [M – H]$^-$ 302.0387, found 302.0385.

Preparation of ^{13}C-3-Hydroxyflavone (^{13}1)

Scheme S5. Synthesis of ^{13}1

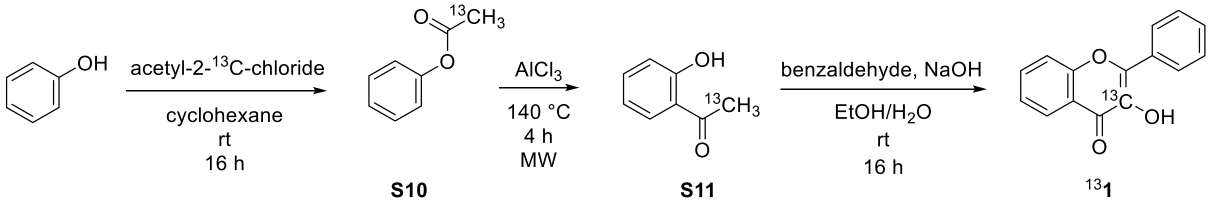

Phenylacetate-2-^{13}C (S10)

To a solution of phenol (250 mg, 2.7 mmol, 1 equiv) in cyclohexane (3 mL) was added acetyl-2-^{13}C-chloride (227 µL, 3.2 mmol, 1.2 equiv). The reaction mixture was stirred at room temperature for 16 h. The reaction mixture was poured into water, and $NaHCO_3$ was added until pH 8 and extracted with CH_2Cl_2 (3 × 50 mL). The combined organic layers were dried over Na_2SO_4, filtered, and evaporated *in vacuo*. The residue was purified by FCC (20:1 petroleum ether/EtOAc) to afford phenyl acetate (**S10**) as a colorless oil (230 mg, 64%). ^{1}H NMR (500 MHz, DMSO-d_6) δ 7.44 – 7.39 (m, 2H), 7.27 – 7.23 (m, 1H), 7.14 – 7.09 (m, 2H), 2.26 (d, J = 130 Hz, 3H) ppm. The NMR data correspond to those in the literature.[12]

1-(2-Hydroxyphenyl)ethan-1-one-2-^{13}C (2′-hydroxyacetophenone, **S11**)

Phenylacetate (**S10**, 225 mg, 1.7 mmol, 1 equiv) was added to $AlCl_3$ (271 mg, 2.0 mmol, 1.2 equiv) in a microwave vial under Ar. The reaction mixture was irradiated in a microwave reactor at 140 °C for 4 h. The reaction mixture was dissolved in CH_2Cl_2 (20 mL) and washed with water (3 × 20 mL). The organic layer was dried with Na_2SO_4, filtered, and evaporated *in vacuo*. The crude product was purified by FCC (20:1 petroleum ether/EtOAc). The desired product **S11** was isolated as a colorless liquid (69 mg, 30%). ^{1}H NMR (500 MHz, DMSO-d_6) δ 11.95 (bs, 1H), 7.89 (dd, J = 8.2, 1.8 Hz, 2H), 7.55 – 7.51 (m, 1H), 6.98 – 6.94 (m, 2H), 2.64 (d, J = 130.0 Hz, 3H) ppm. The NMR data correspond to those in the literature.[12]

3-Hydroxy-2-phenyl-4*H*-chromen-4-one-3-^{13}C (^{13}C-3-hydroxyflavone, ^{13}**1**)

Compound **S11** (60 mg, 0.4 mmol, 1 equiv) was dissolved in EtOH (3 mL), and NaOH (88.14 mg, 2.2 mmol, 5 equiv) dissolved in 1 mL H_2O was added. After 5 min, benzaldehyde (135 µL, 1.3 mmol, 3 equiv) was added and the reaction mixture was stirred for 4 h at rt. H_2O_2 (30% aqueous solution, 225 µL, 2.2 mmol, 5 equiv) was added, and the reaction mixture was stirred for 16 h at rt. The reaction mixture was acidified to pH 6.5 with 1 M HCl (aqueous solution). The resulting precipitate was centrifuged, washed with water, and dried *in vacuo*. ^{13}C-3-hydroxyflavone (^{13}**1**) was isolated as a yellow solid (12.7 mg, 13%). ^{1}H NMR (500 MHz, DMSO-d_6) δ 9.63 (dm, 1H), 8.25–8.21 (m, 2H), 8.13 (dd, J = 7.9, 1.5 Hz, 1H), 7.85 – 7.76 (m, 2H), 7.60 – 7.55 (m, 2H), 7.55 – 7.44 (m, 2H) ppm. ^{13}C{^{1}H} NMR (126 MHz, DMSO-d_6) δ 173.2 (d, J = 57.7 Hz), 154.6, 148.6, 145.6 (d, J = 89.8 Hz), 144.8, 139.1, 133.8, 131.3, 129.2, 128.5, 127.7, 124.8, 124.6, 121.3 (d, J = 14 Hz), 118.5 ppm. The NMR data correspond to those in the literature.[13] HRMS (APCI–) *m/z*: calcd. for $C_{14}{}^{13}CH_9O_3$ $[M - H]^-$ 238.0591, found 238.0590.

Preparation of 2-(3,4-dihydroxyphenyl)-3-hydroxy-4*H*-chromen-4-one-3-^{13}C (^{13}C-3,3′,4′-trihydroxy flavonol, ^{13}3)

Scheme S6. Preparation of ^{13}3.

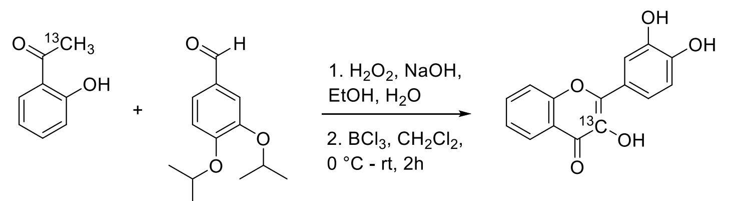

2′-Hydroxyacetophenone (**S11**, 60 mg, 0.4 mmol, 1 equiv), 3,4-diisopropoxybenzaldehyde (**S1**, 98 mg, 0.4 mmol, 1 equiv), and NaOH (53 mg, 1.3 mmol, 3 equiv) were dissolved in an EtOH/H_2O mixture (4 mL, 3:1). The reaction was carried out in a microwave reactor at 65 °C for 40 min. H_2O_2 (30 % aqueous solution, 135 µL, 1.3 mmol, 3 equiv) was added, and the reaction was carried out in the microwave reactor at 40 °C for 20 min. The reaction mixture was poured into water and extracted with EtOAc (3 × 10 mL). The combined organic layers were dried over Na_2SO_4, filtered, and evaporated *in vacuo*. The residue was dissolved in 3 mL of dry CH_2Cl_2, and BCl_3 (1 M in CH_2Cl_2, 1.3 mL, 1.3 mmol, 3 equiv) was added. The reaction mixture was heated at 40 °C for 2 h. The reaction mixture was cooled to 0 °C, and an excess of methanol was added. The reaction mixture was evaporated, and the residue was purified by preparative HPLC chromatography (ASAHIPAK, methanol, 5 mL/min, isocratic). Flavonol ^{13}**3** was isolated as a brown solid (14 mg, 14%). ^{1}H NMR (500 MHz, DMSO-d_6) δ 9.59 (s, 1H), 9.30 (s, 2H), 8.10 (dd, J = 7.9, 1.6 Hz, 1H), 7.81 – 7.74 (m, 2H), 7.73–7.69 (m, 1H), 7.61 (dd, J = 8.5, 0.6 Hz, 1H), 7.45 (m, 1H), 6.90 (d, J = 8.5 Hz, 1H) ppm. ^{13}C{^{1}H} NMR (126 MHz, DMSO-d_6) δ 172.7 (d, J = 58.9 Hz), 154.3, 147.6, 145.7 (d, J = 89.0 Hz), 145.1, 137.9, 133.4, 124.7, 124.3, 122.3, 121.3 (d, J = 13.0 Hz), 120.0, 118.2, 115.6, 115.2 ppm. The NMR data

correspond to the previously reported data for the unlabeled compound.[6] HRMS (APCI–) *m/z*: calcd. for $C_{14}{}^{13}CH_9O_5$ [M – H]⁻ 270.0489, found 270.0491.

Preparation of 3-hydroxy-2-phenyl-4*H*-benzo[g]chromen-4-one-3-^{13}C (^{13}7) and 3-hydroxy-2-phenyl-4*H*-benzo[g]chromene-4-thione-3-^{13}C (^{13}8)

Scheme S7. Synthesis of ^{13}7 and ^{13}8

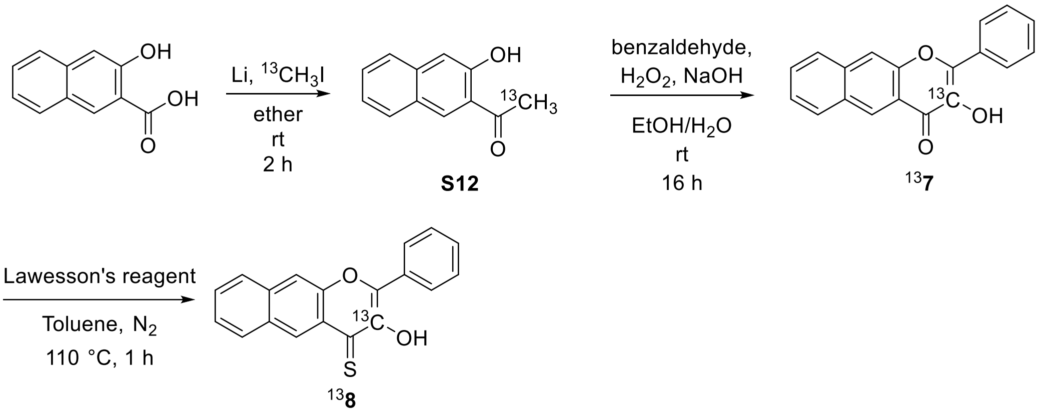

1-(3-Hydroxynaphthalen-2-yl)ethan-1-one-2-^{13}C (S12)

Granular Li (44.4 mg, 6.3 mmol, 2 equiv) was suspended in ether (3 mL) under an Ar atmosphere in a Schlenk flask. ^{13}C-Methyl iodide (197 µL, 3.2 mmol, 1 equiv) dissolved in 0.5 mL ether was added dropwise. The reaction was carried out until reflux ceased and a white precipitate was formed. 3-Hydroxy-2-naphthoic acid (200 mg, 1.0 mmol, 1 equiv) dissolved in ether (1 mL) was added dropwise. The reaction was stirred at rt for 2 h. The reaction was quenched with water. After the decomposition of the excess of Li, the reaction was extracted with CH_2Cl_2 (3 × 10 mL). The organic fraction was dried over Na_2SO_4, filtered, and evaporated *in vacuo*. The residue was purified by FCC (1:1 petroleum ether/EtOAc). Product **S12** was isolated as a yellow solid (75 mg, 40%). ^{1}H NMR (500 MHz, DMSO-d_6) δ 11.32 (bs., 1H), 8.61 (s, 1H), 7.98 (d, *J* = 8.2 Hz, 1H), 7.76 (d, *J* = 7.6 Hz, 1H), 7.55 (ddd, *J* = 8.4, 6.9, 1.2 Hz, 1H), 7.37 (ddd, *J* = 8.2, 6.9, 1.1 Hz, 1H), 7.29 (s, 1H), 2.78 (d, *J* = 128.3 Hz, 3H) ppm. The NMR data correspond to the previously reported data for the unlabeled compound.[14]

3-Hydroxy-2-phenyl-4*H*-benzo[g]chromen-4-one-3-^{13}C (^{13}7)

Compound **S12** (75 mg, 0.6 mmol, 1 equiv) was dissolved in EtOH (3 mL), and NaOH (110 mg, 2.8 mmol, 5 equiv) dissolved in 1 mL H_2O was added. After 5 min, benzaldehyde (168 µL, 1.7 mmol, 3 equiv) was added. The reaction mixture was stirred for 4 h at rt. H_2O_2 (30% aqueous solution, 281 µL, 2.7 mmol, 5 equiv) was added, and the reaction mixture was stirred for 16 h at rt. The reaction mixture was poured into water and extracted with EtOAc (3 × 10 mL). The organic fraction was dried over Na_2SO_4, filtered, and evaporated *in vacuo*. The residue was purified by FCC (85:10:5:1 chloroform/toluene/acetone/formic acid), and compound ^{13}**7** was isolated as a brown solid (20 mg, 13%). ^{1}H NMR (500 MHz, DMSO-d_6) δ 9.62 (bs, 1H), 8.85 (s, 1H), 8.33 (s, 1H), 8.31 – 8.27 (m, 2H), 8.26 (d, *J* = 8.4 Hz, 1H), 8.08 (d, *J* = 8.54 Hz, 1H), 7.73 – 7.65 (m, 1H), 7.64 – 7.50 (m, 4H) ppm. ^{13}C{^{1}H} NMR (126 MHz, DMSO-d_6) δ 174.0 (d, *J* = 58.3 Hz), 150.9, 146.0 (d, *J* = 90.0 Hz), 137.8, 135.4, 131.5, 130.1, 129.4, 129.4, 128.8,

128.6, 127.9, 127.2, 126.0, 125.9, 120.9 (d, J = 13.0 Hz), 114.2 ppm. The NMR data correspond to the previously reported data for the unlabeled compound.[14] HRMS (APCI–) *m/z*: calcd. for $C_{18}{}^{13}CH_{11}O_3$ $[M - H]^-$ 288.0747, found 288.0745.

3-Hydroxy-2-phenyl-4*H*-benzo[g]chromene-4-thione-3-^{13}C (^{13}8)

Compound ^{13}**7** (8.8 mg, 0.03 mmol, 1 equiv) was dissolved in dry toluene (0.6 mL) under an N_2 atmosphere. Lawesson's reagent (15 mg, 0.04 mmol, 1.2 equiv) was added, and the reaction mixture was heated to 110 °C under magnetic stirring. After 1 h, the reaction mixture changed its color from orange to dark red, and no starting material was detected on TLC (CH_2Cl_2/hexane 1:2). The reaction mixture was cooled down and purified by column chromatography (silica, CH_2Cl_2/hexane 6:1), and compound ^{13}**8** was isolated as a bright red solid (4.7 mg, 51%). ^{1}H NMR (300 MHz, $CDCl_3$) δ 9.17 (s, 1H), 8.64 (br, 1H), 8.52–8.48 (m, 2H), 8.15 (s, 1H), 8.12 (s, 1H), 7.98–7.95 (m, 1H), 7.67–7.52 (m, 5H) ppm. ^{13}C NMR (126 MHz, $CDCl_3$) δ 189.7, 189.3, 147.4, 147.3, 145.2, 145.1, 141.2, 135.6, 131.2, 129.6, 129.1, 128.9, 128.8, 127.1, 126.2, 114.6, 109.10. The NMR data correspond to the previously reported data for the unlabeled compound.[15] HRMS (APCI+) *m/z*: calcd. for $C_{18}{}^{13}CH_{13}O_2S$ $[M + H]^+$ 306.0664, found 306.0664.

Preparation of 2-(3,4-dimethoxyphenyl)-3-hydroxy-4*H*-chromen-4-one-3-^{13}C (^{13}9)

Scheme S8. Preparation of ^{13}9.

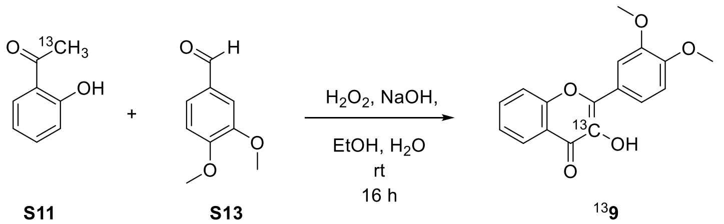

3,4-Dimethoxybenzaldehyde (S13)

3,4-Dihydroxybenzaldehyde (1.0 g, 7 mmol, 1 equiv) and K_2CO_3 (5.0 g, 35 mmol, 5 equiv) were dissolved in DMF (20 mL). Iodomethane (4.5 mL, 70 mmol, 10 equiv) was added. The reaction mixture was stirred at room temperature for 16 h. The reaction mixture was poured into water and extracted with CH_2Cl_2 (3 × 100 mL). The organic phase was washed with water (3 × 100 mL), brine (3 × 100 mL), dried over Na_2SO_4, and evaporated *in vacuo*. The residue was purified by FCC (2:1 cyclohexane/EtOAc) to give compound **S13** as a colorless oil (1.1 g, 98%). ^{1}H NMR (500 MHz, DMSO-d_6) δ 9.84 (s, 1H), 7.56 (dd, J = 8.2, 2.1 Hz, 1H), 7.39 (d, J = 1.8 Hz, 1H), 7.18 (d, J = 8.2 Hz, 1H), 3.87 (s, 3H), 3.83 (s, 3H) ppm. The NMR data correspond to those in the literature.[16]

2-(3,4-Dimethoxyphenyl)-3-hydroxy-4H-chromen-4-one-3-^{13}C (^{13}9)

2′-Hydroxyacetophenone (**S11**, 60 mg, 0.4 mmol, 1 equiv) was dissolved in EtOH (3 mL), and NaOH (88.1 mg, 2.2 mmol, 5 equiv) dissolved in 1 mL H_2O was added. After 5 min, 3,4-dimethoxybenzaldehyde (**S13**, 220 mg, 1.3 mmol, 3 equiv) was added, and the reaction mixture was stirred for 16 h at rt. H_2O_2 (30% aqueous solution, 225 µL, 2.2 mmol, 5 equiv) was added, and the reaction mixture was stirred for 4 h at rt. The reaction mixture was acidified to pH 6.5

with 1 M HCl (aqueous solution). The precipitate was centrifuged, washed with water, and dried *in vacuo*. Flavonol ^{13}**9** was isolated as a yellow solid (24 mg, 20%). ^{1}H NMR (500 MHz, DMSO-d_6) δ 9.49 (s, 1H), 8.13–8.08 (m, 1H), 7.88 (dd, J = 8.5, 2.1 Hz, 1H), 7.83 – 7.79 (m, 3H), 7.48 – 7.44 (m, 1H), 7.16 (d, J = 8.9 Hz, 1H), 3.85 (s, 6H) ppm. ^{13}C{^{1}H} NMR (126 MHz, DMSO-d_6) δ 172.6 (d, J = 59.0 Hz), 154.4 (d, J = 1.9 Hz), 150.3, 148.4, 145.5 (d, J = 90.0 Hz), 138.3, 133.5, 124.7, 124.5, 123.6 (d, J = 2.0 Hz), 121.5, 121.3 (d, J = 14.0 Hz), 118.4, 111.5, 110.9, 55.7, 55.6 ppm. The NMR data correspond to the previously reported data for the unlabeled compound.[6] HRMS (APCI–) *m/z*: calcd. for $C_{16}{}^{13}CH_{13}O_5$ $[M - H]^-$ 298.0802, found 298.0799.

3. NMR spectra

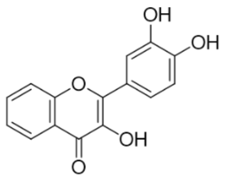

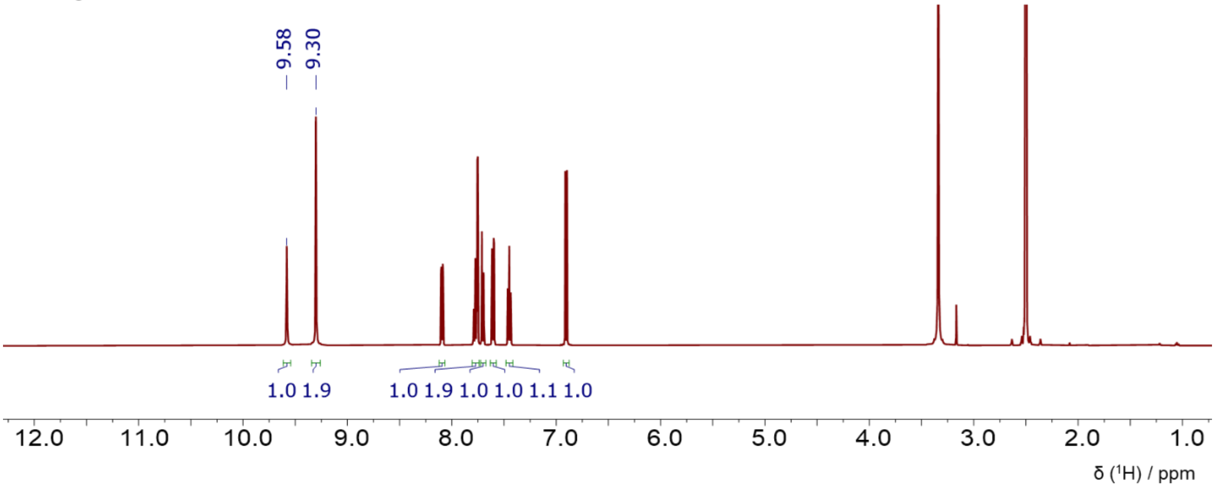

Figure S1. ^{1}H NMR (500 MHz, DMSO-d_6, 25 °C) spectrum of **3**.

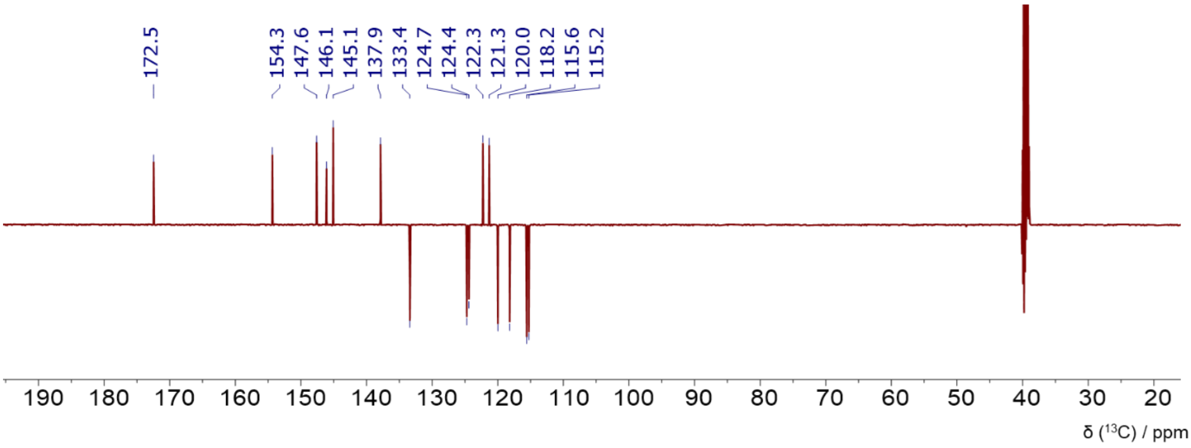

Figure S2. ^{13}C APT NMR (126 MHz, DMSO-d_6, 25 °C) spectrum of **3**.

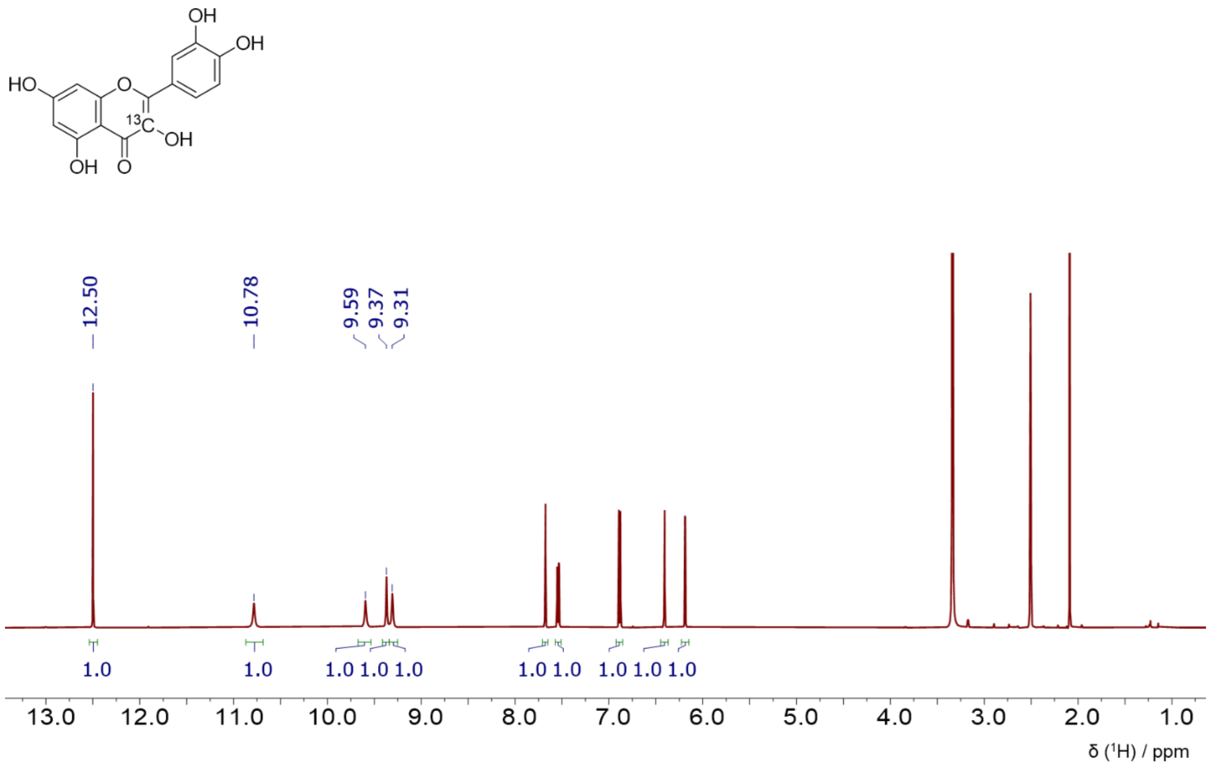

Figure S3. ^{1}H NMR (500 MHz, DMSO-d_6, 25 °C) spectrum of ^{13}**2**.

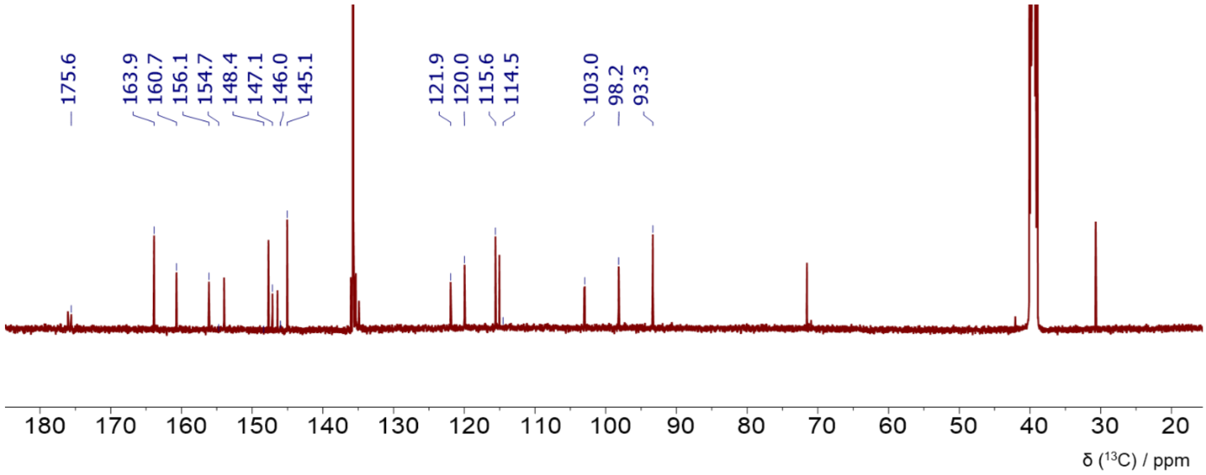

Figure S4. ^{13}C NMR (126 MHz, DMSO-d_6, 25 °C) spectrum of ^{13}**2**.

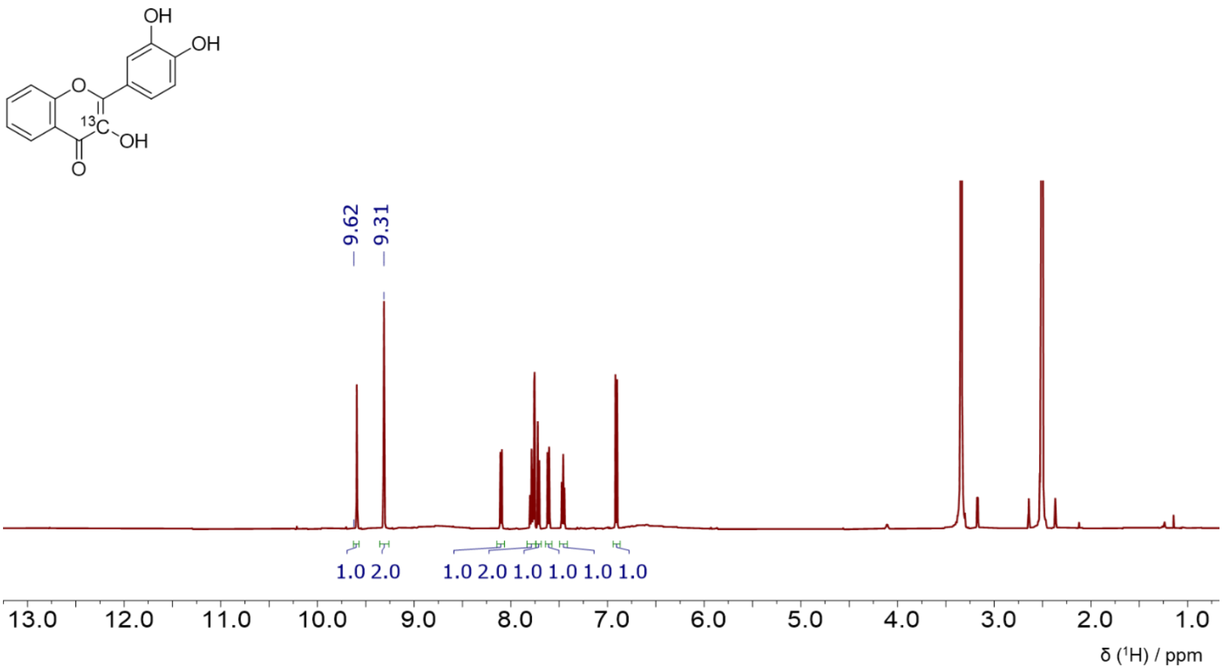

Figure S5. ^{1}H NMR (500 MHz, DMSO-d_6, 25 °C) spectrum of ^{13}**3**.

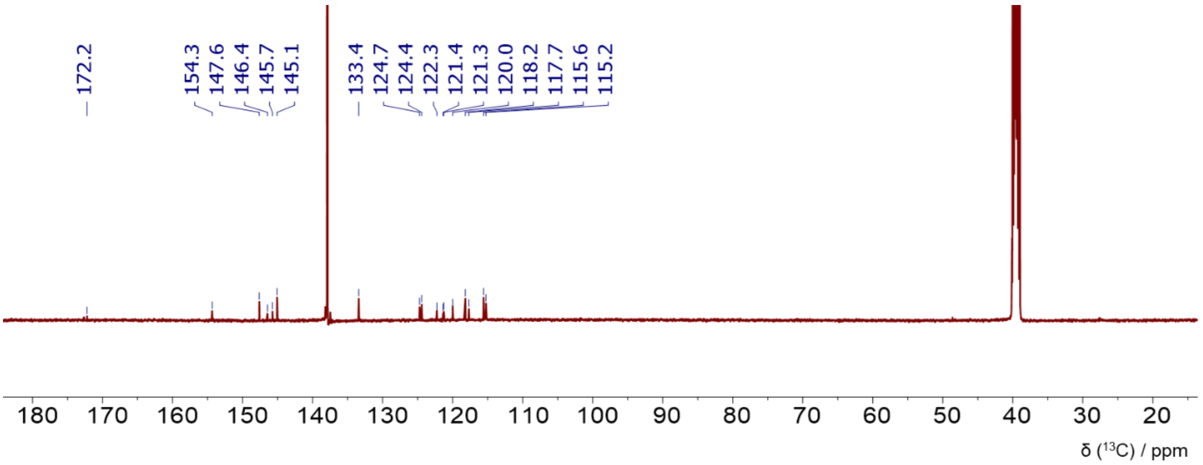

Figure S6. ^{13}C NMR (126 MHz, DMSO-d_6, 25 °C) spectrum of ^{13}**3**.

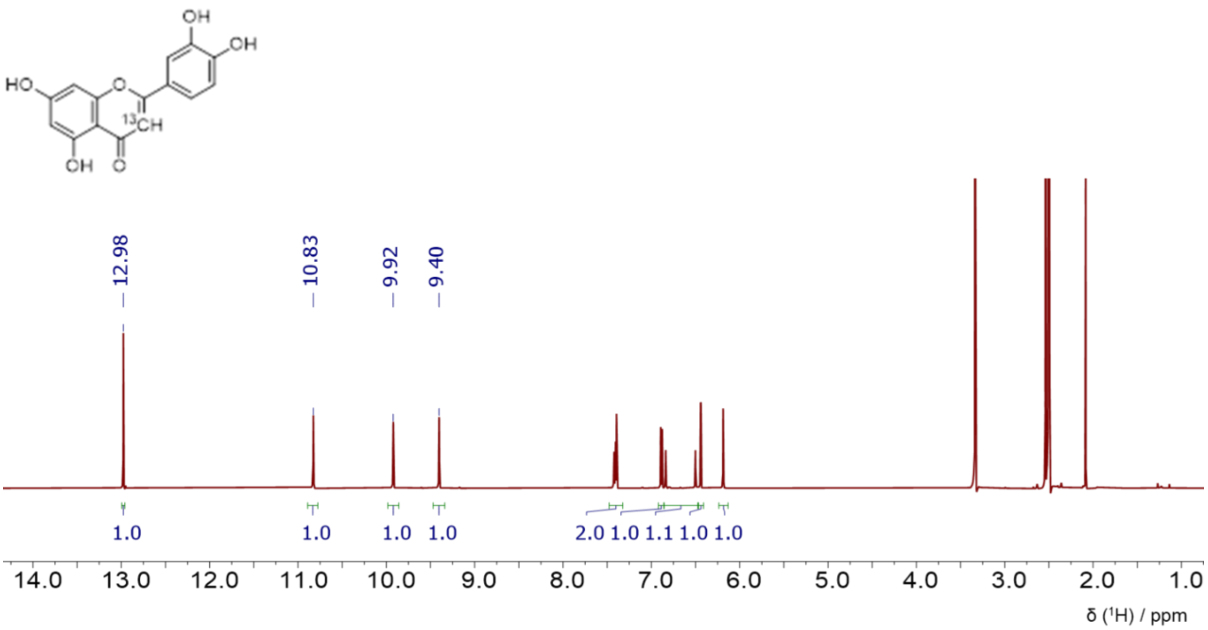

Figure S7. ^{1}H NMR (500 MHz, DMSO-d_6, 25 °C) spectrum of ^{13}**5**.

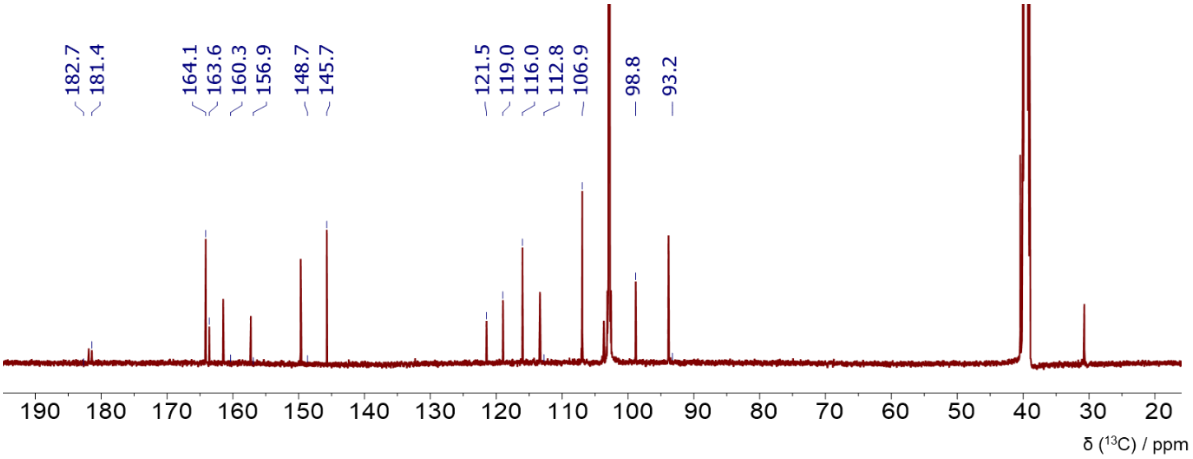

Figure S8. ^{13}C NMR (126 MHz, DMSO-d_6, 25 °C) spectrum of ^{13}**5**.

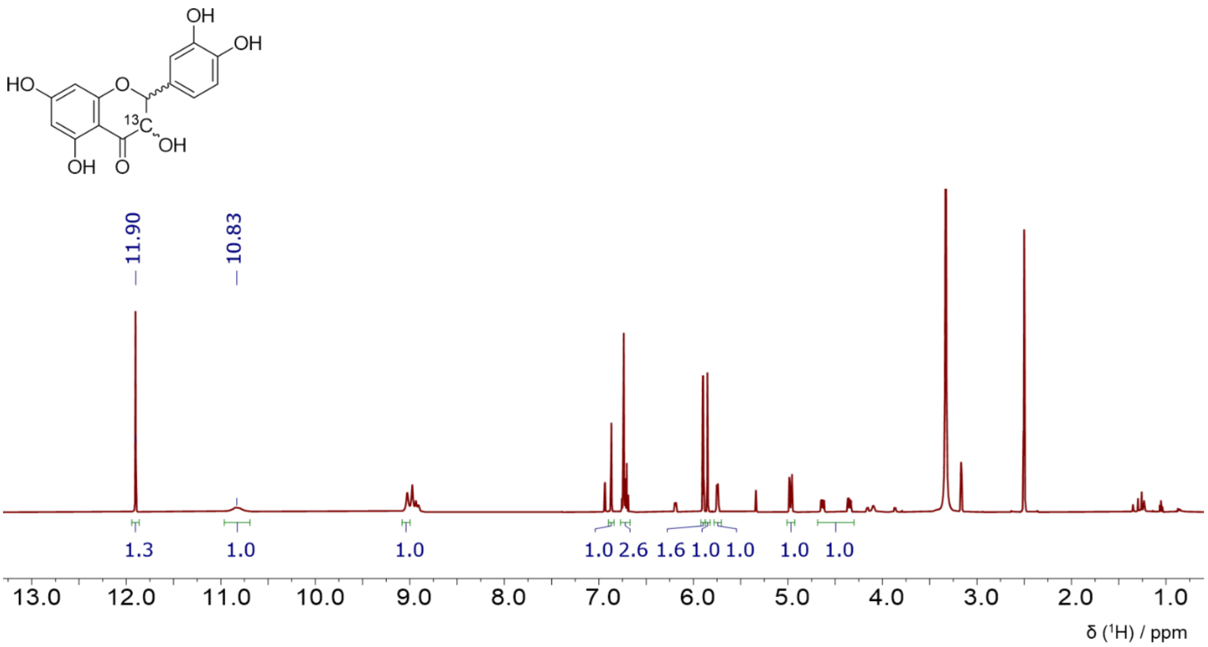

Figure S9. ^{1}H NMR (500 MHz, DMSO-d_6, 25 °C) spectrum of ^{13}**6**.

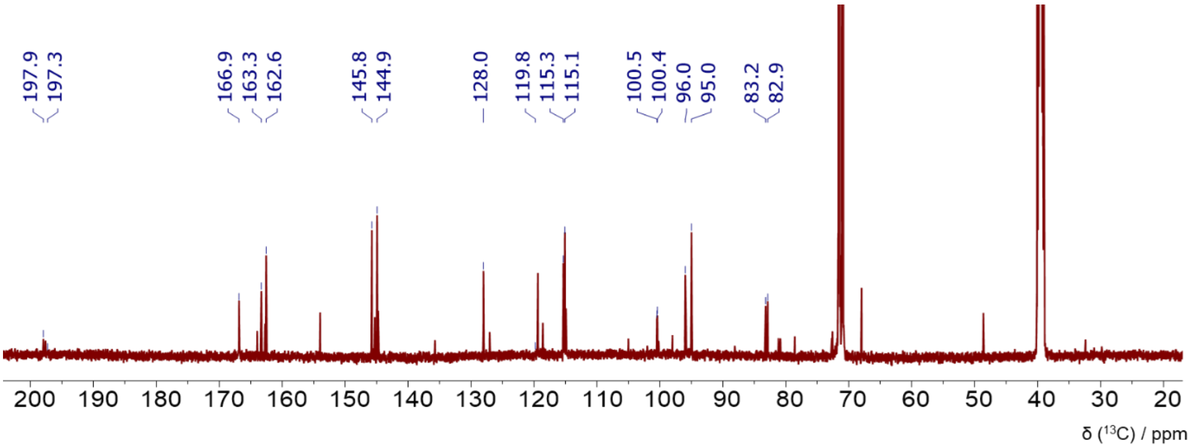

Figure S10. ^{13}C NMR (126 MHz, DMSO-d_6, 25 °C) spectrum of ^{13}**6**.

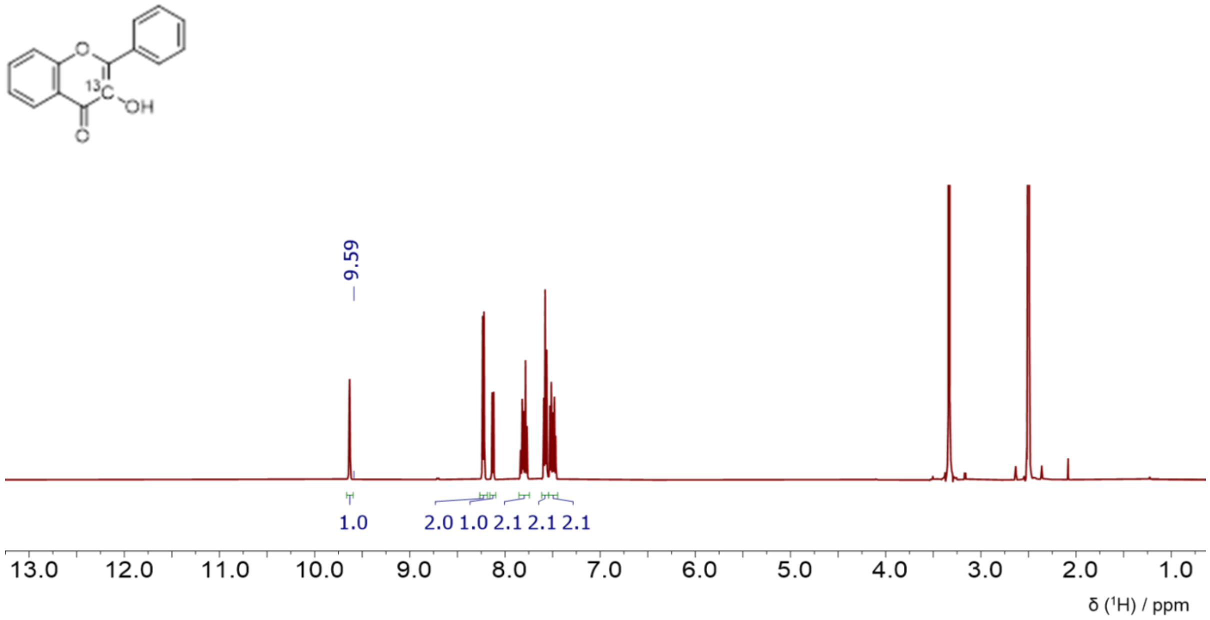

Figure S11. ^{1}H NMR (500 MHz, DMSO-d_6, 25 °C) spectrum of ^{13}**1**.

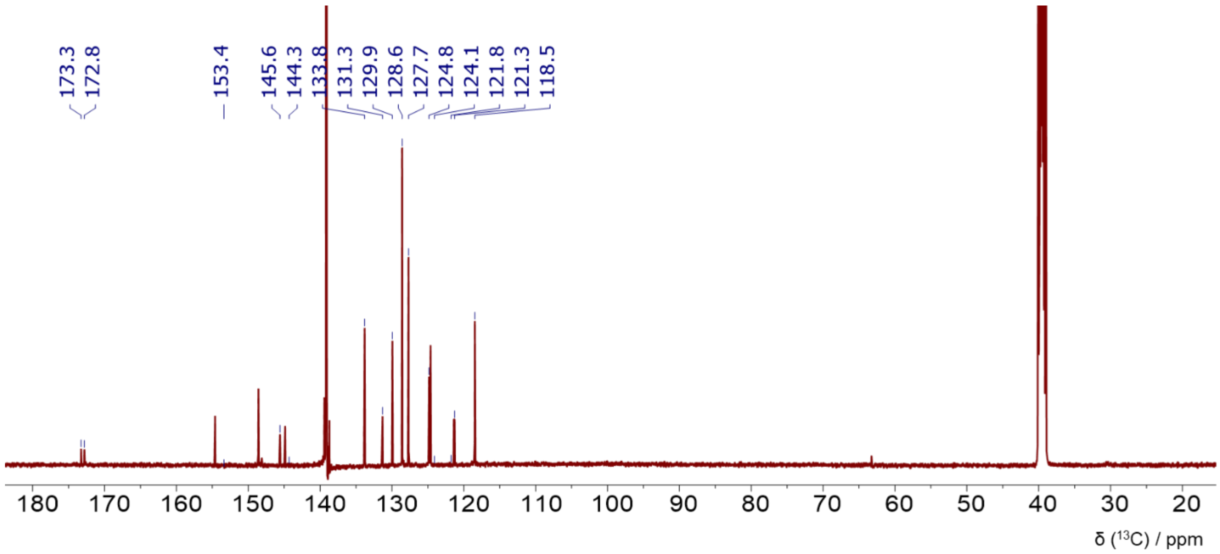

Figure S12. ^{13}C NMR (126 MHz, DMSO-d_6, 25 °C) spectrum of ^{13}**1**.

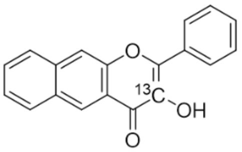

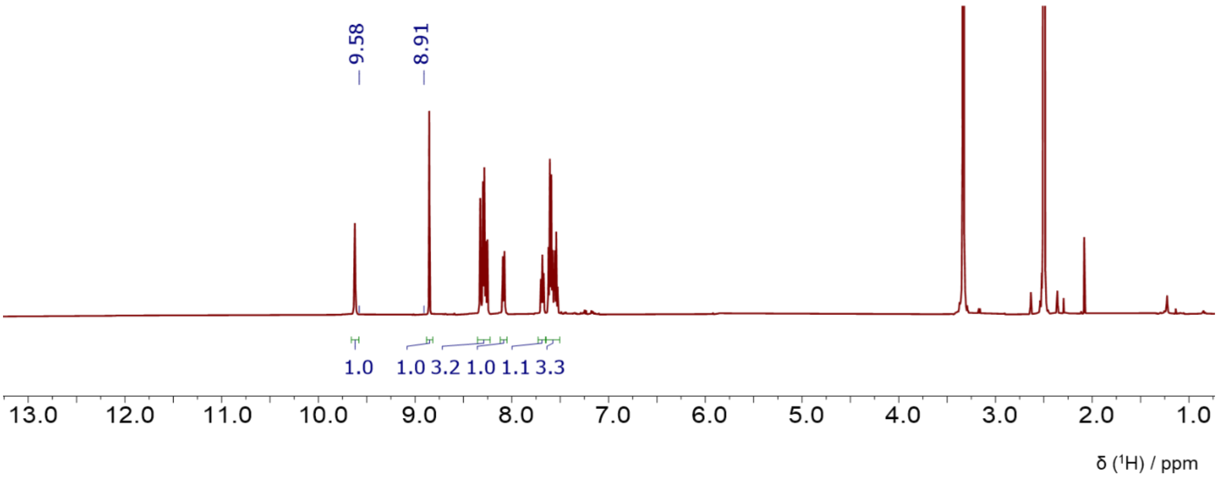

Figure S13. ^{1}H NMR (500 MHz, DMSO-d_6, 25 °C) spectrum of ^{13}**7**.

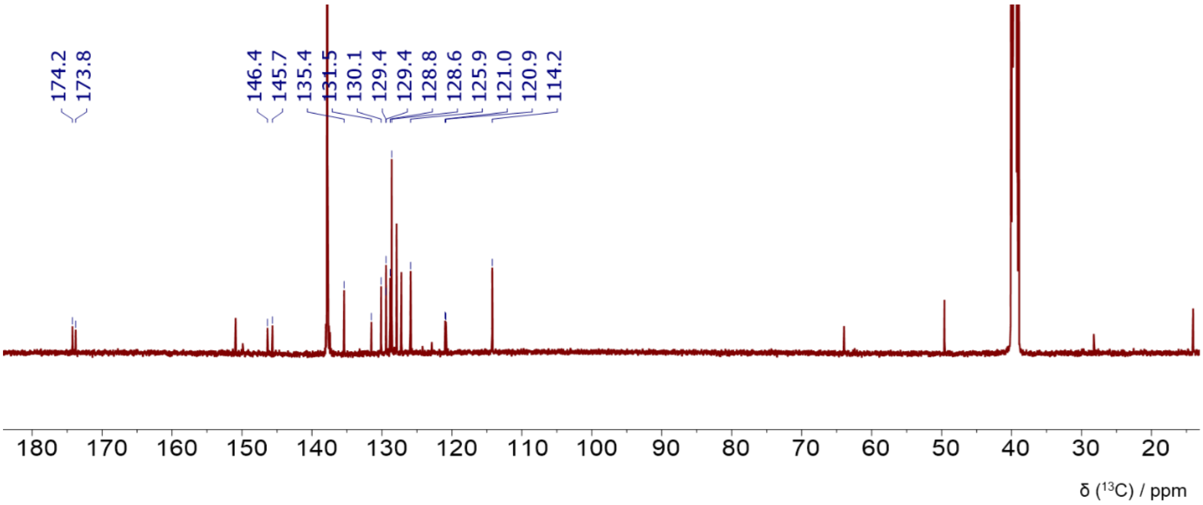

Figure S14. ^{13}C NMR (126 MHz, DMSO-d_6, 25 °C) spectrum of ^{13}**7**.

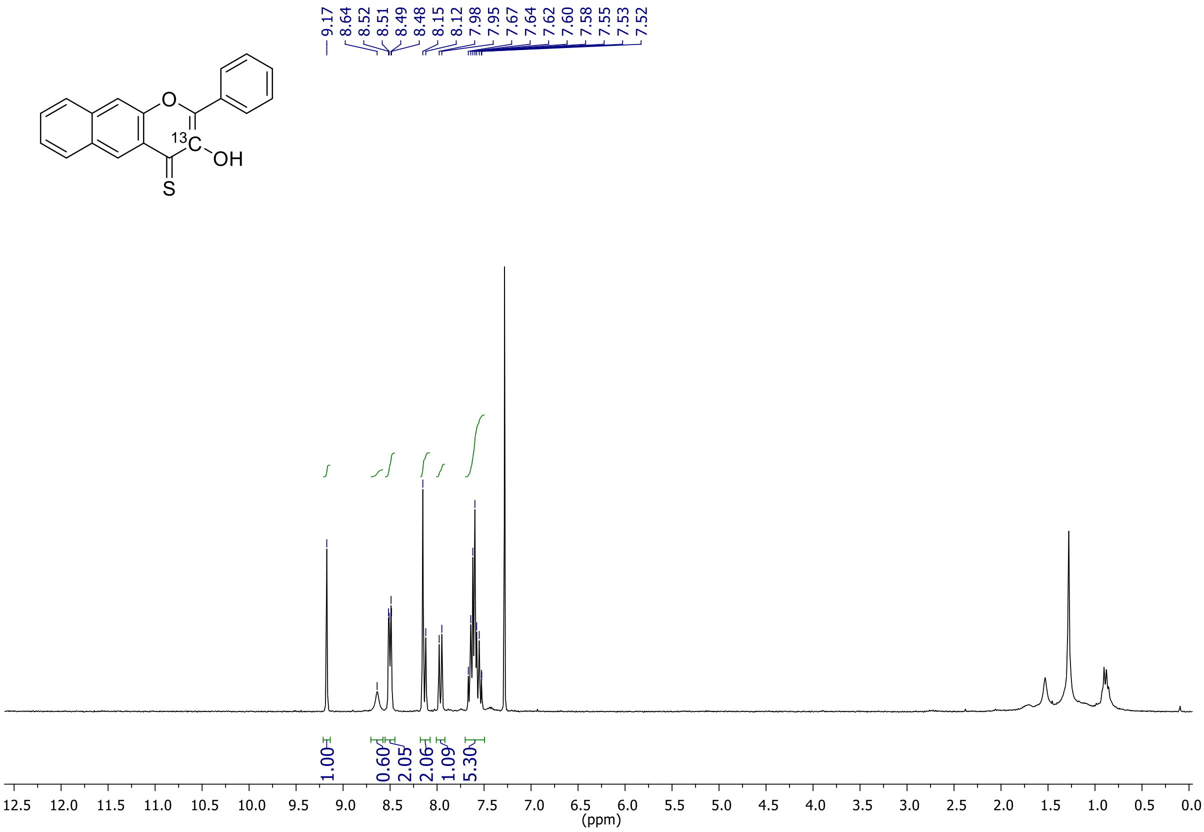

Figure S15. ^{1}H NMR (300 MHz, $CDCl_3$, 25 °C) spectrum of ^{13}**8**.

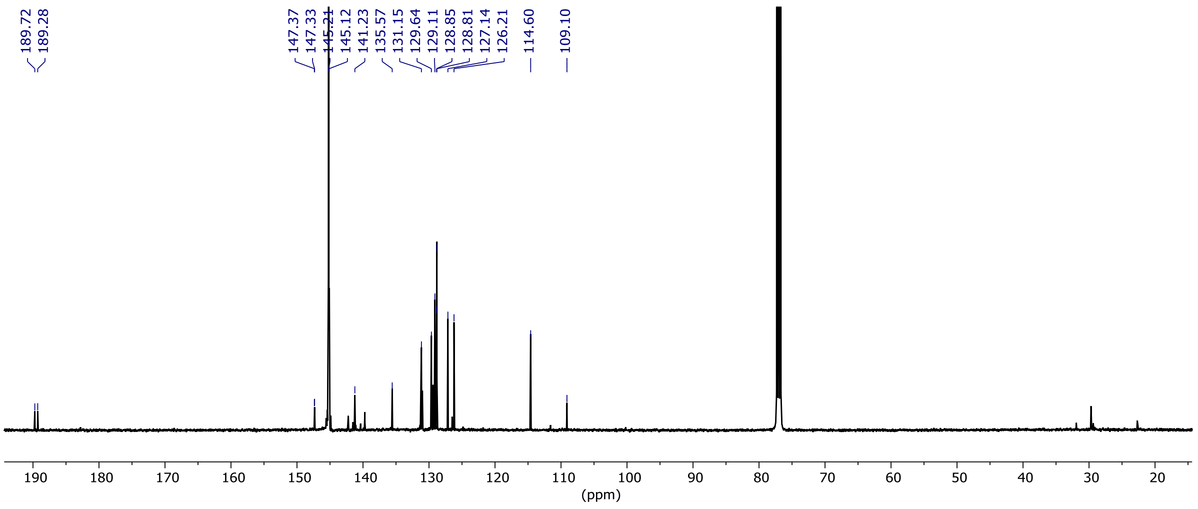

Figure S16. ^{13}C NMR (126 MHz, $CDCl_3$, 25 °C) spectrum of ^{13}**8**.

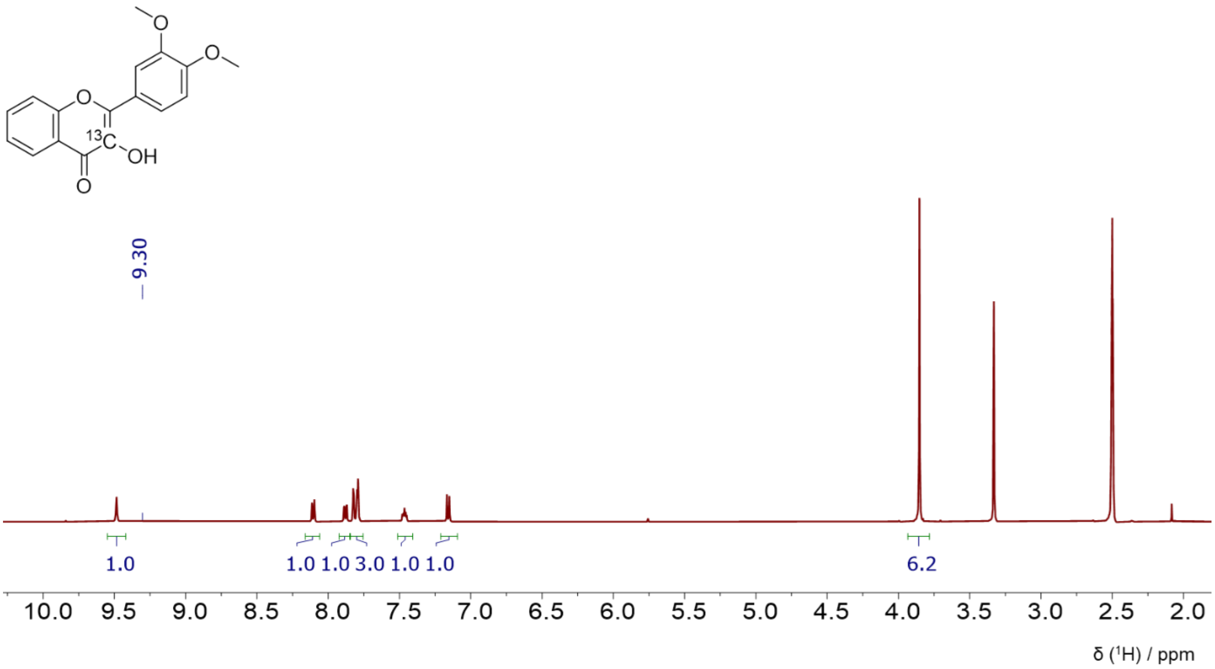

Figure S17. ^{1}H NMR (500 MHz, DMSO-d_6, 25 °C) spectrum of ^{13}**9**.

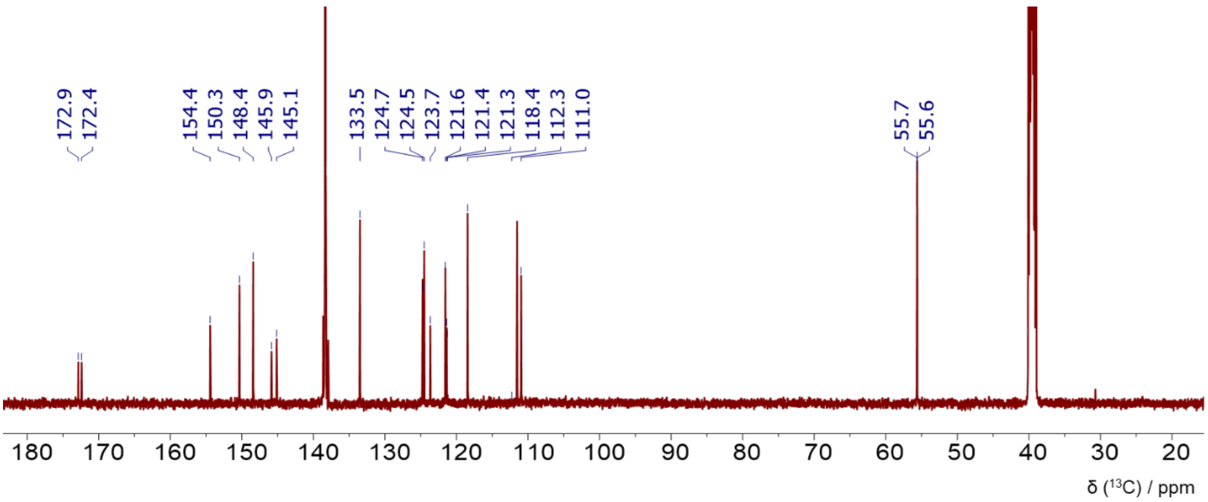

Figure S18. ^{13}C NMR (126 MHz, DMSO-d_6, 25 °C) spectrum of ^{13}**9**.

4. HPLC Analyses

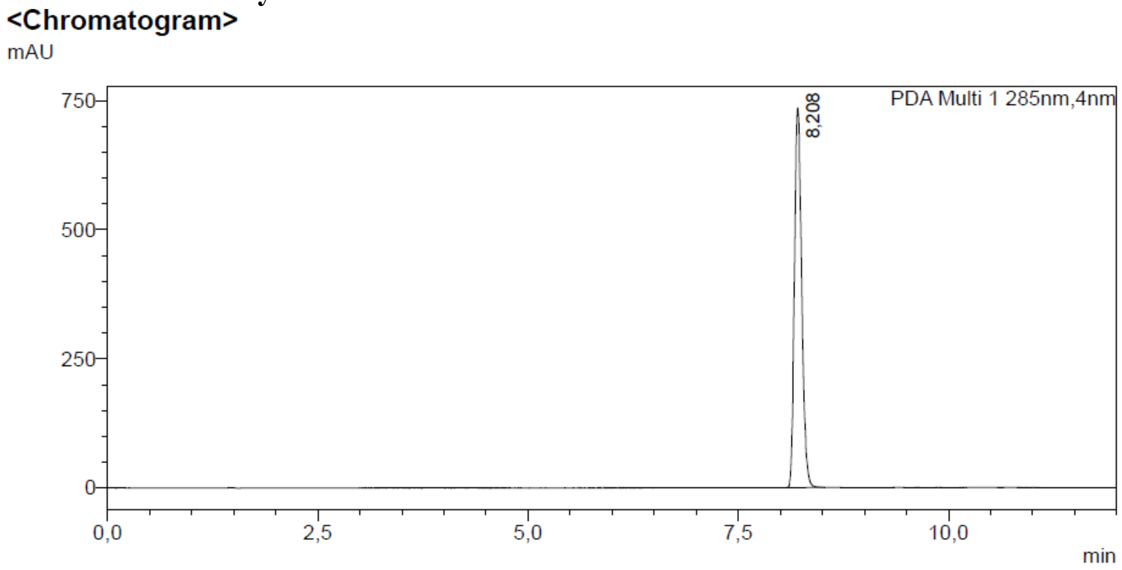

Figure S19. HPLC chromatogram of [13]**1** detected at 285 nm. Arion Polar C18, 0.4 mL/min, 25 °C. Product peak at 8.208 min, purity 100%.

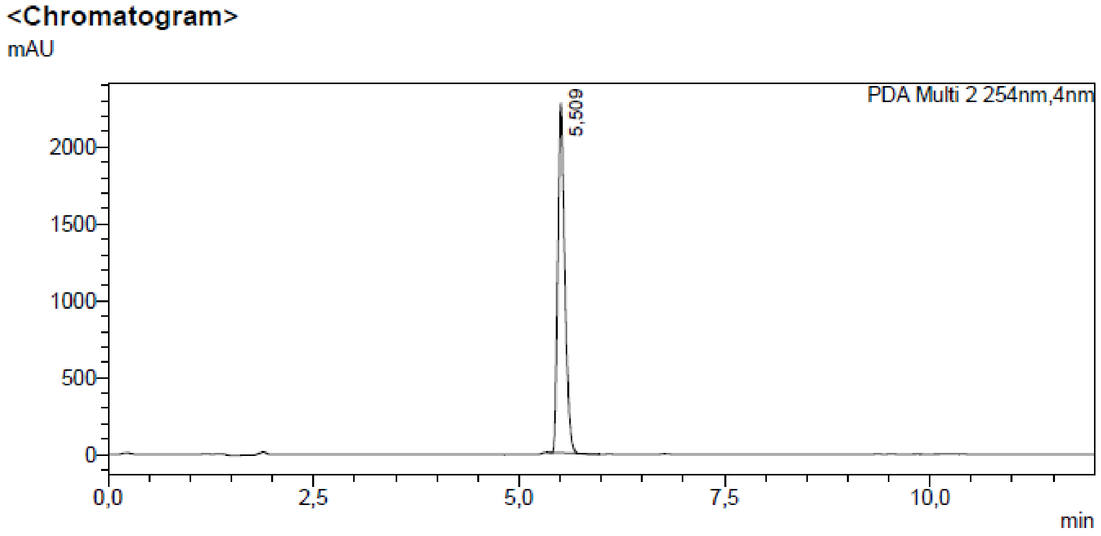

Figure S20. HPLC chromatogram of [13]**2** detected at 254 nm. Arion Polar C18, 0.4 mL/min, 25 °C. Product peak at 5.509 min, purity 100%.

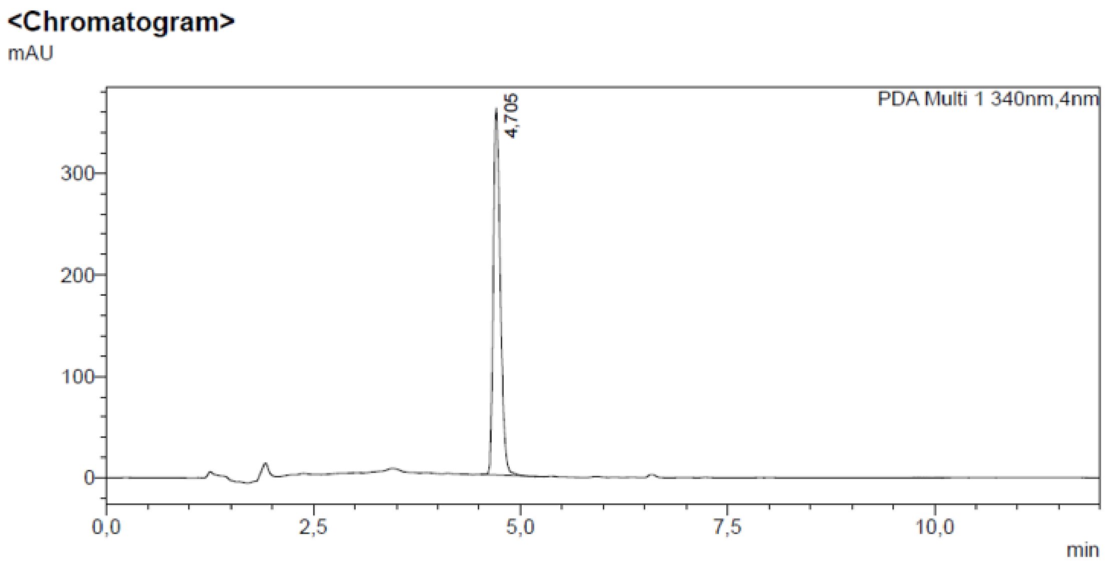

Figure S21. HPLC chromatogram of ^{13}**3** detected at 340 nm. Arion Polar C18, 0.4 mL/min, 25 °C. Product peak at 4.705 min, purity 100%.

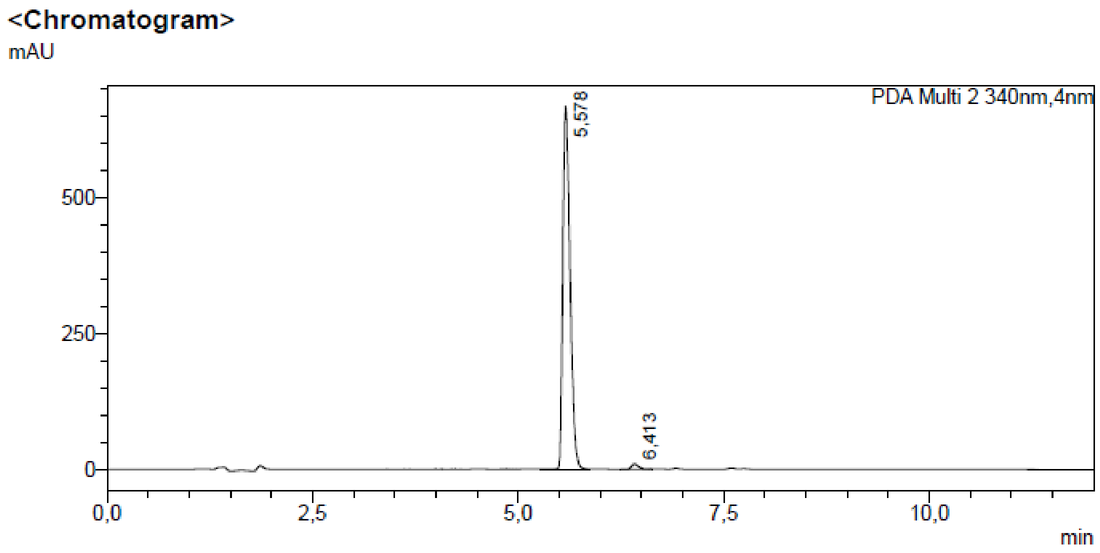

Figure S22. HPLC chromatogram of ^{13}**5** detected at 340 nm. Arion Polar C18, 0.4 mL/min, 25 °C. Product peak at 5.578 min, purity 99%.

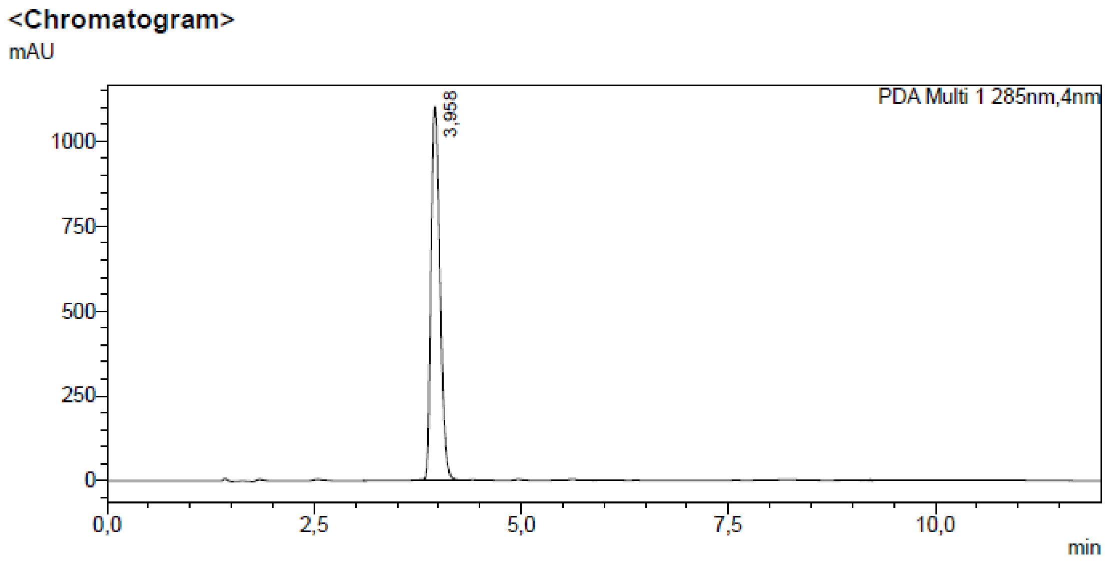

Figure S23. HPLC chromatogram of [13]**6** detected at 285 nm. Arion Polar C18, 0.4 mL/min, 25 °C. Product peak at 3.958 min, purity 100%.

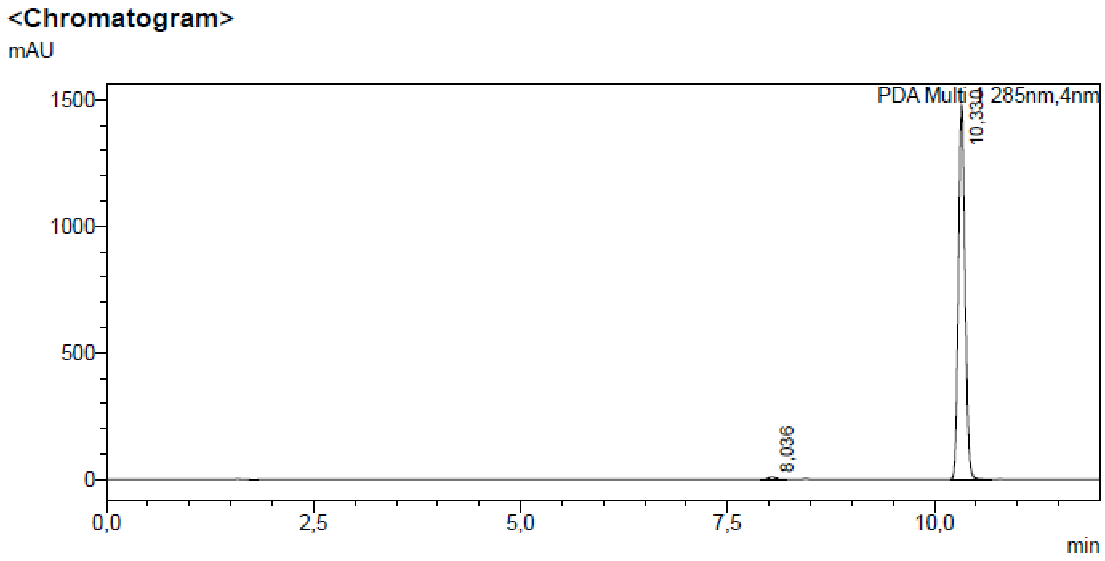

Figure S24. HPLC chromatogram of [13]**7** detected at 285 nm. Arion Polar C18, 0.4 mL/min, 25 °C. Product peak at 10.330 min, purity 99%.

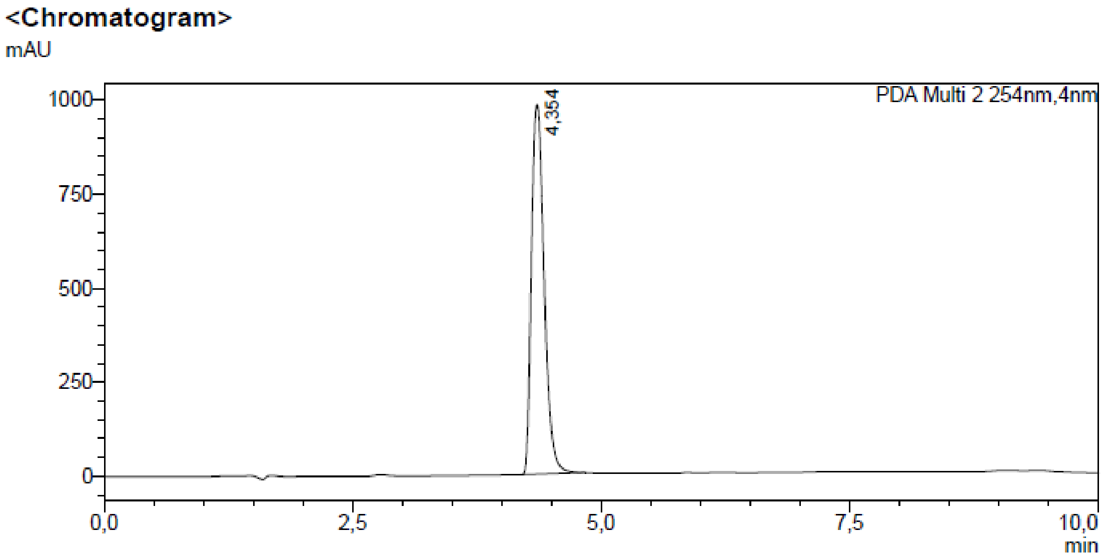

Figure S25. HPLC chromatogram of [13]**9** detected at 254 nm. Arion Polar C18, 50-100% AcN, 0.4 mL/min, 25 °C. Product peak at 4.354 min, purity 100%.

5. HR-MS

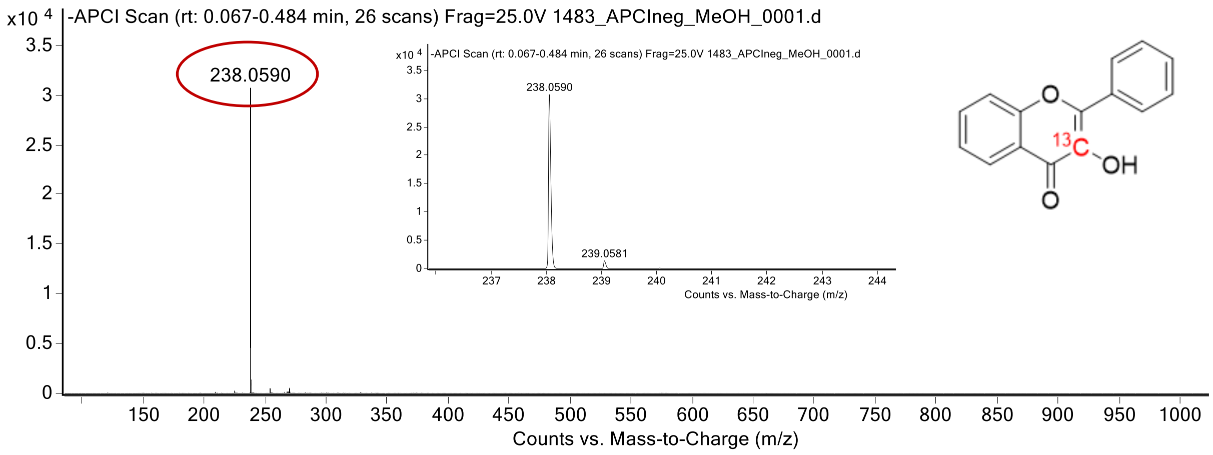

Figure S26. HRMS (APCI–): [13]**1**.

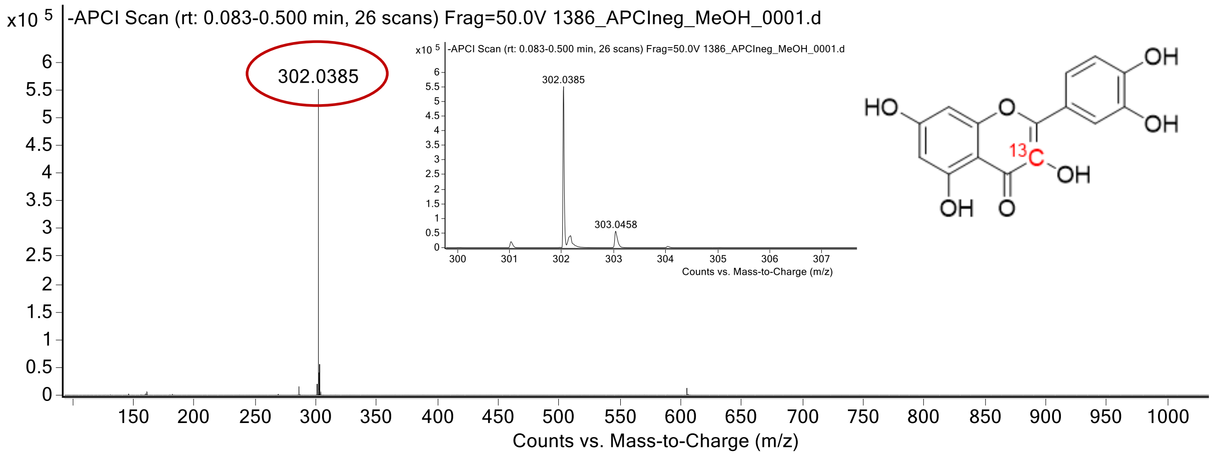

Figure S27. HRMS (APCI–): [13]**2**.

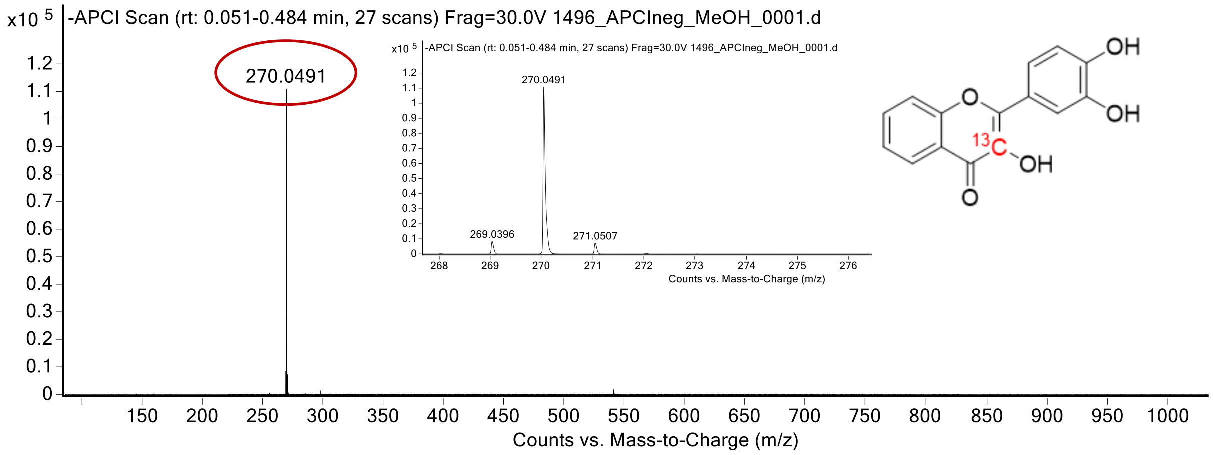

Figure S28. HRMS (APCI–): [13]**3**.

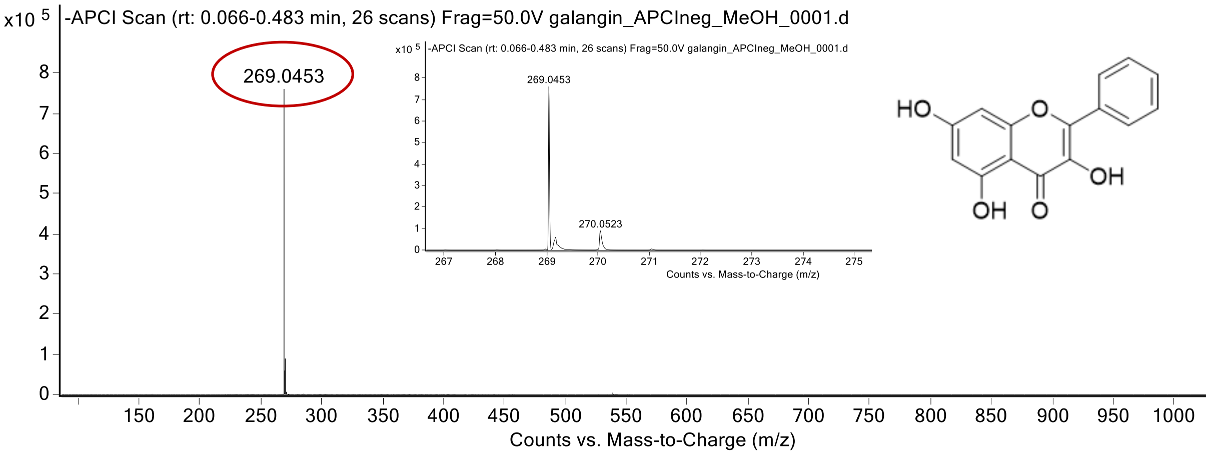

Figure S29. HRMS (APCI–): **4**.

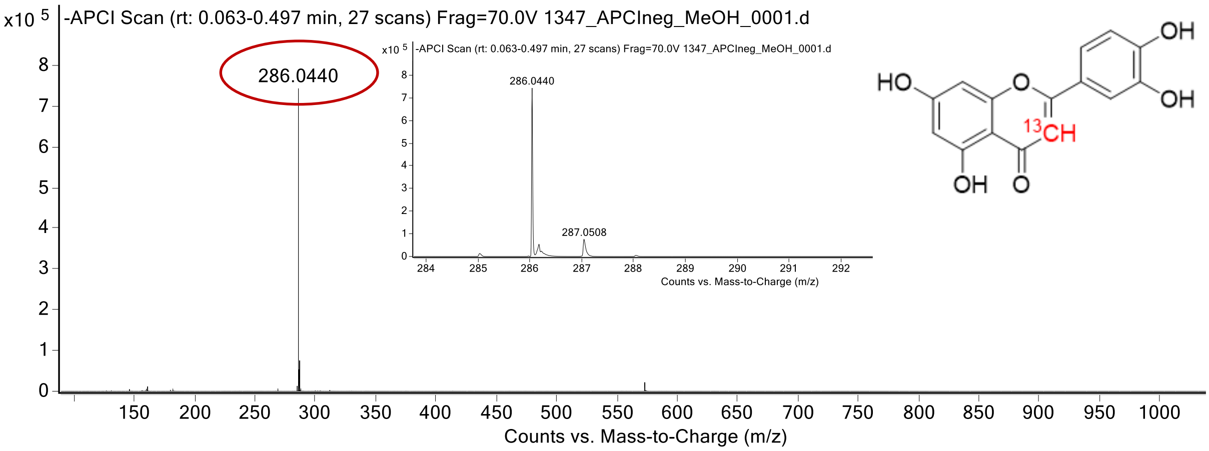

Figure S30. HRMS (APCI–): ^{13}**5**.

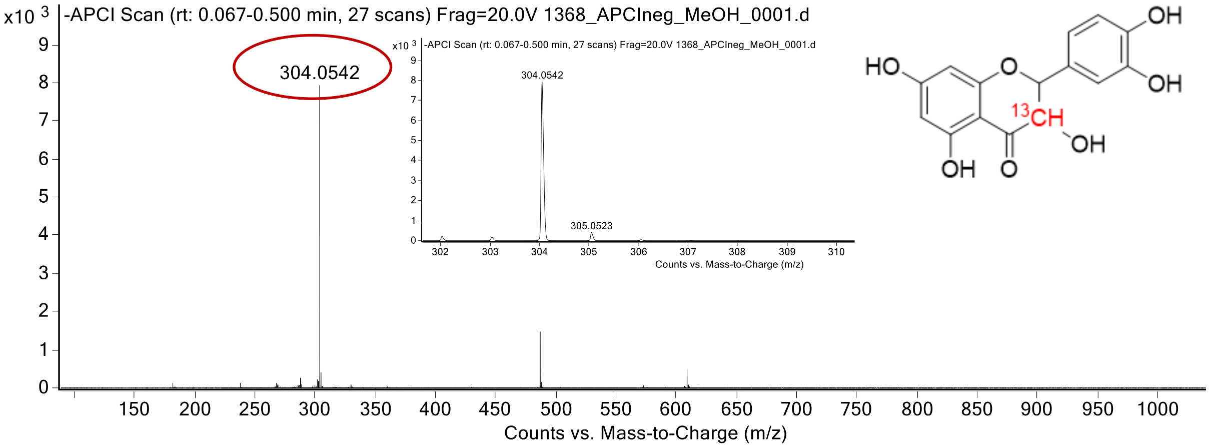

Figure S31. HRMS (APCI–): ^{13}**6**.

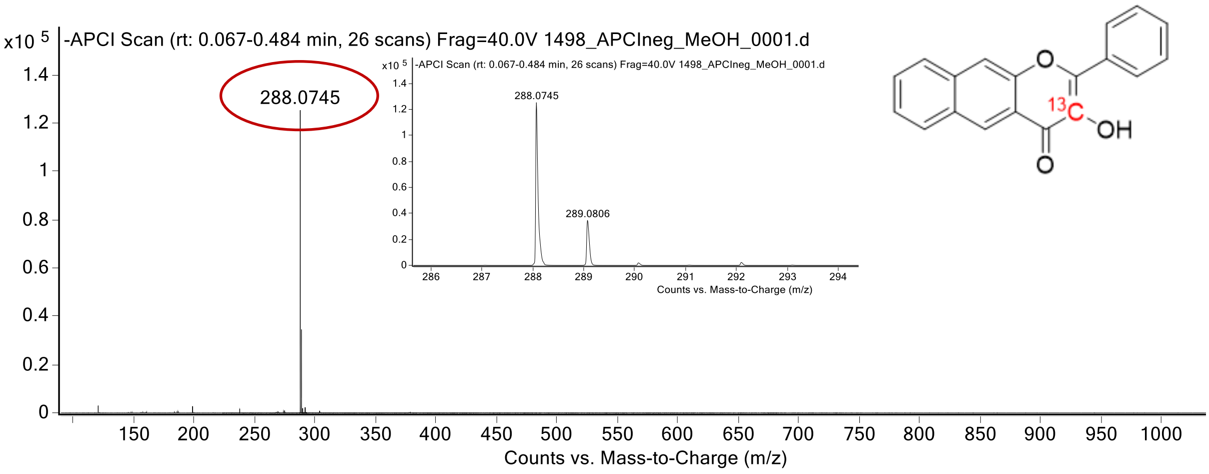

Figure S32. HRMS (APCI–): [13]**7**.

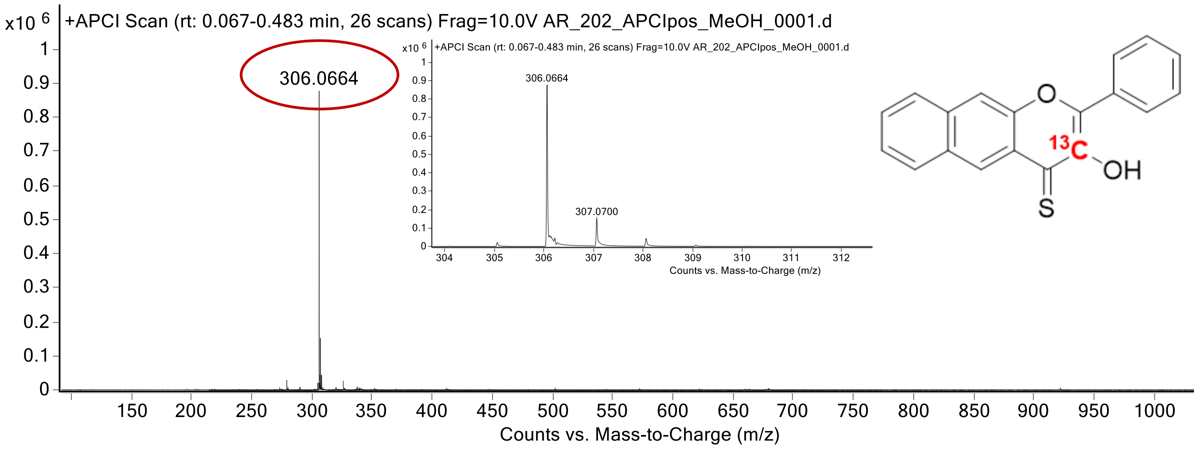

Figure S33. HRMS (APCI+): [13]**8**.

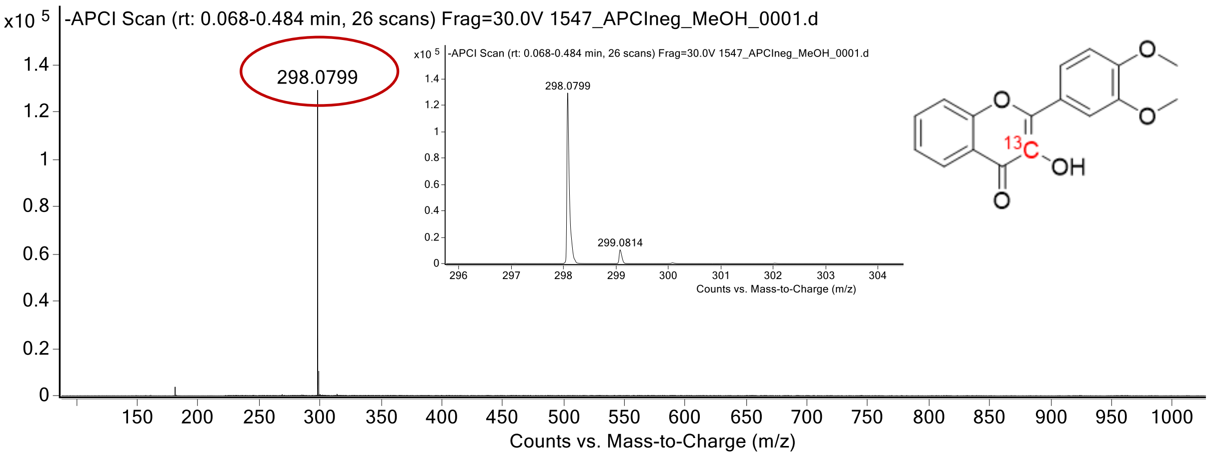

Figure S34. HRMS (APCI–): [13]**9**.

6. UV-Vis Absorption Spectra

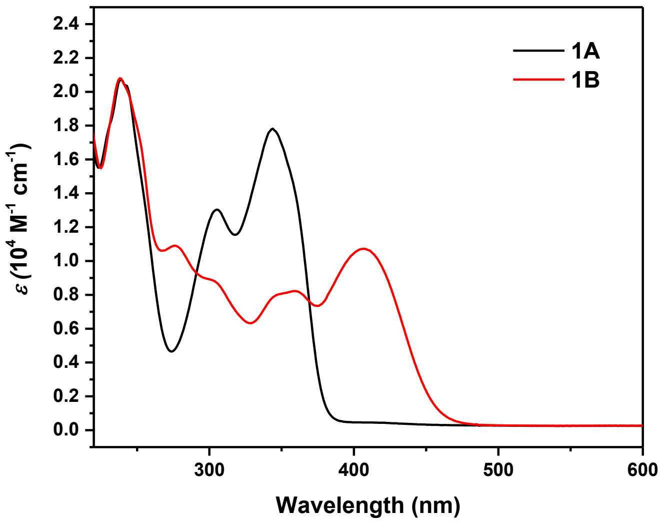

Figure S35. UV-Vis spectra of **1** (100 μM) in methanol (black) or basic methanol (red; 6 equiv of $NaOCH_3$).

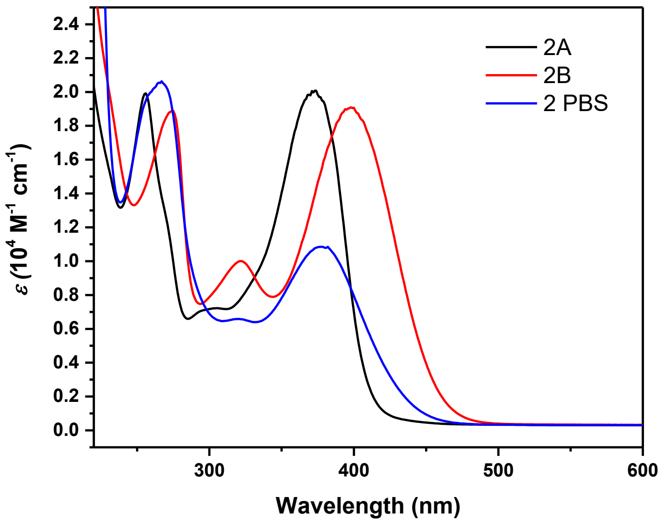

Figure S36. UV-Vis spectra of **2** (100 μM) in methanol (black), basic methanol (red) or PBS solution (blue; 5 % of DMSO, pH = 7.4, c = 10 mM, I = 100 mM).

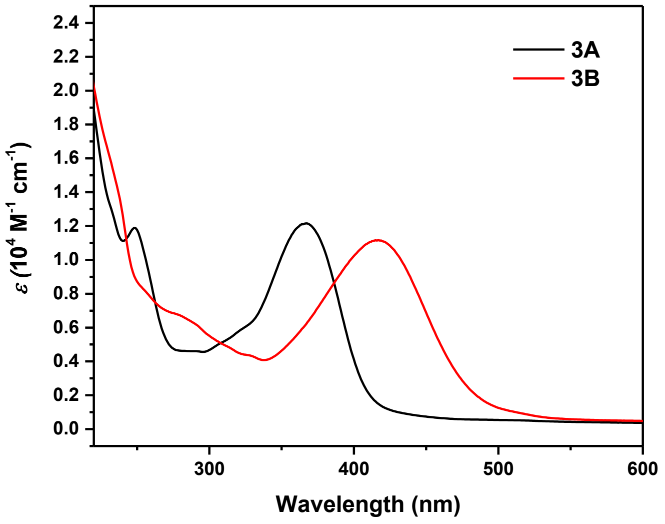

Figure S37. UV-Vis spectra of **3** (100 µM) in methanol (black) or basic methanol (red; 6 equiv of $NaOCH_3$).

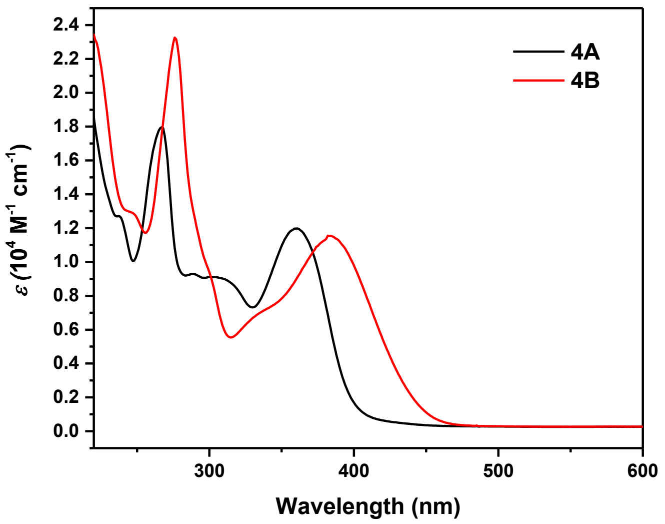

Figure S38. UV-Vis spectra of **4** (100 µM) in methanol (black) or basic methanol (red; 6 equiv of $NaOCH_3$).

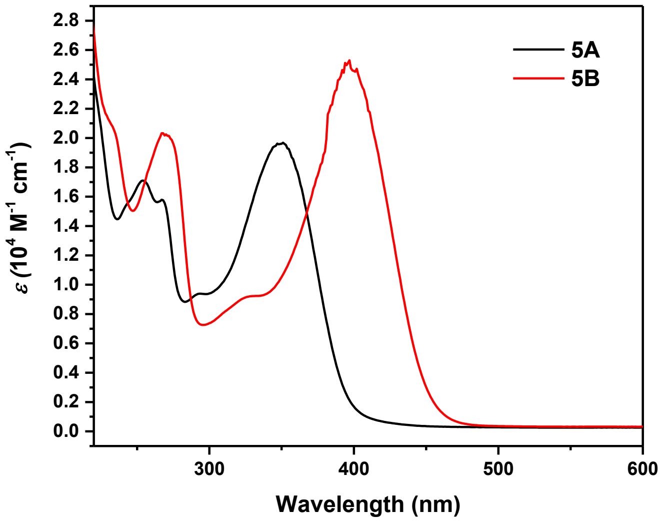

Figure S39. UV-Vis spectra of **5** (100 μM) in methanol (black) or basic methanol (red; 6 equiv of $NaOCH_3$).

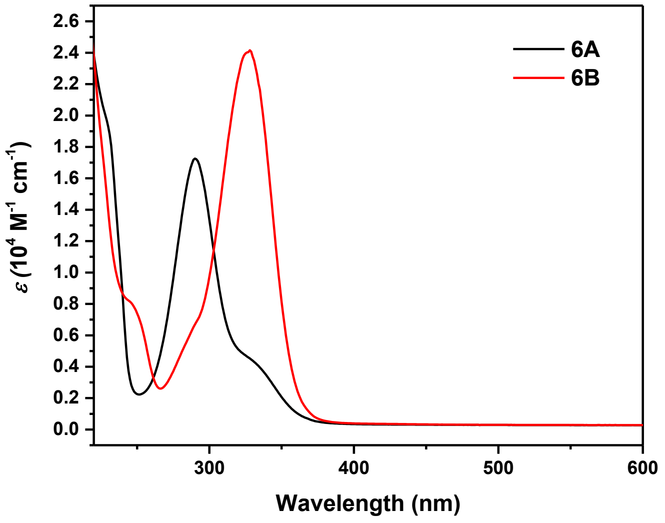

Figure S40. UV-Vis spectra of **6** (100 μM) in methanol (black) or basic methanol (red; 6 equiv of $NaOCH_3$).

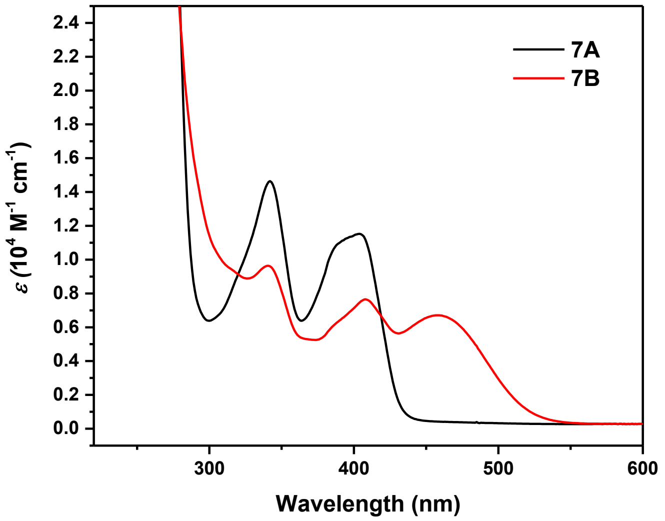

Figure S41. UV-Vis spectra of **7** (100 µM) in methanol (black) or basic methanol (red; 6 equiv of $NaOCH_3$).

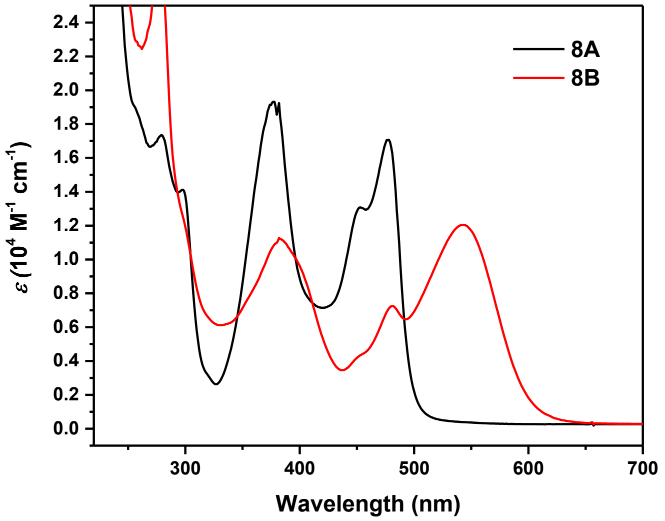

Figure S42. UV-Vis spectra of **8** (100 µM) in methanol (black) or basic methanol (red; 6 equiv of $NaOCH_3$).

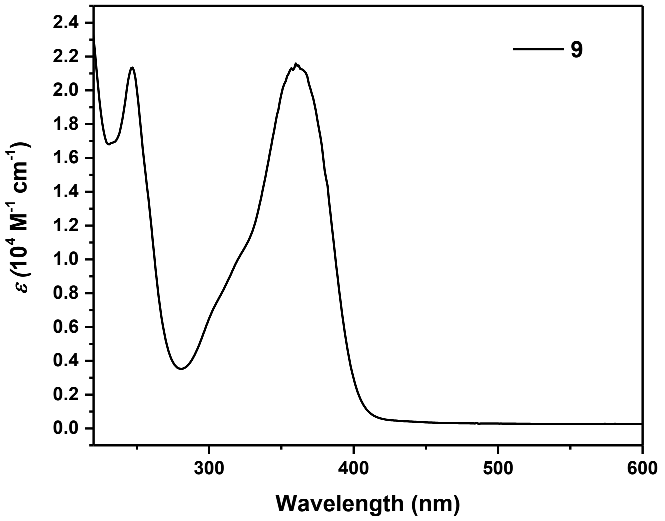

Figure S43. UV-Vis spectra of **9** (100 μM) in methanol.

7. Photoproduct Analyses

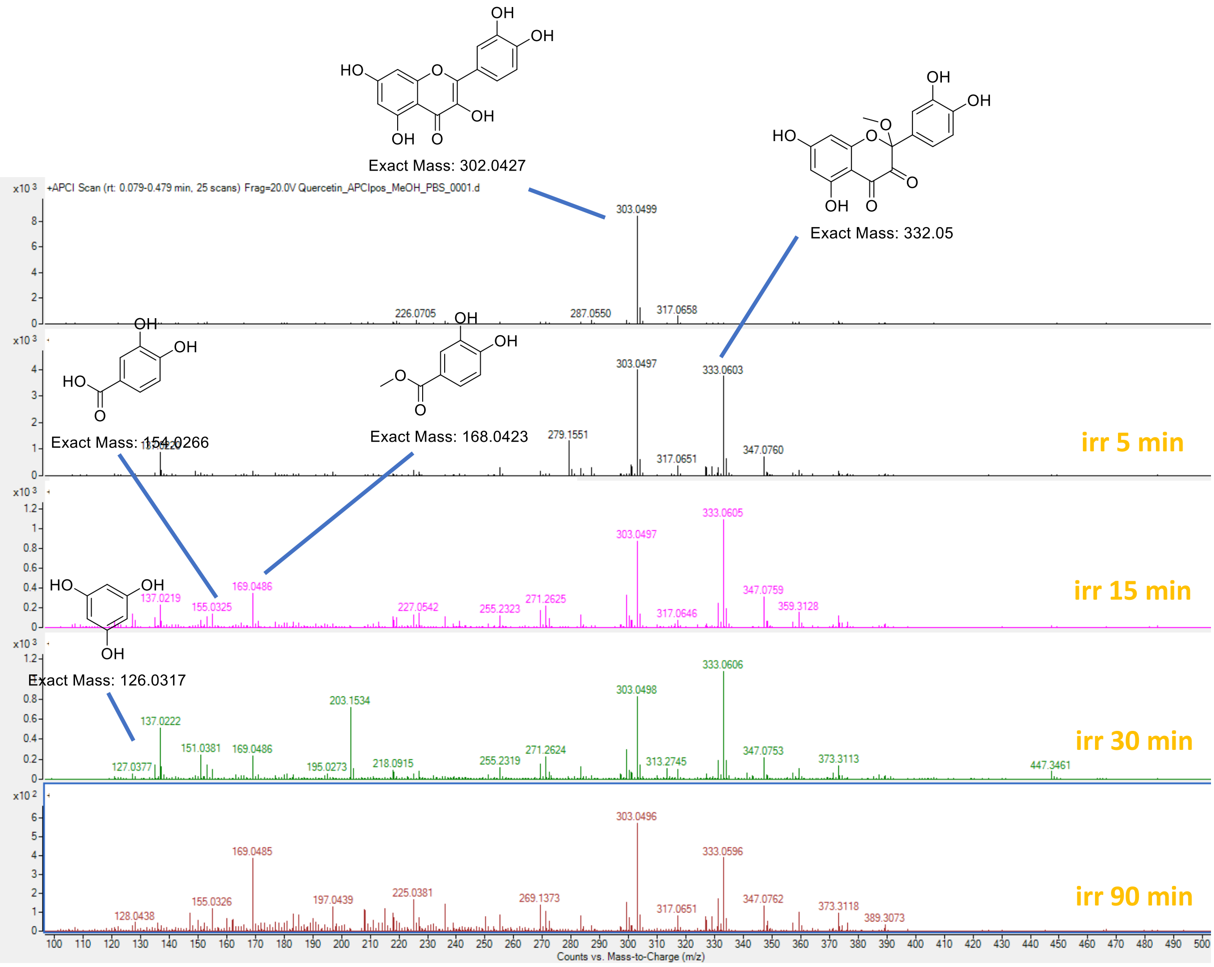

Figure S44. HR-MS (ESI+) of a solution of **2** (100 µM) and Rose Bengal (5 µM) in methanol-PBS (3:7 v/v) after irradiation at 545 nm for different amount of time.

8. Transient Absorption Spectroscopy

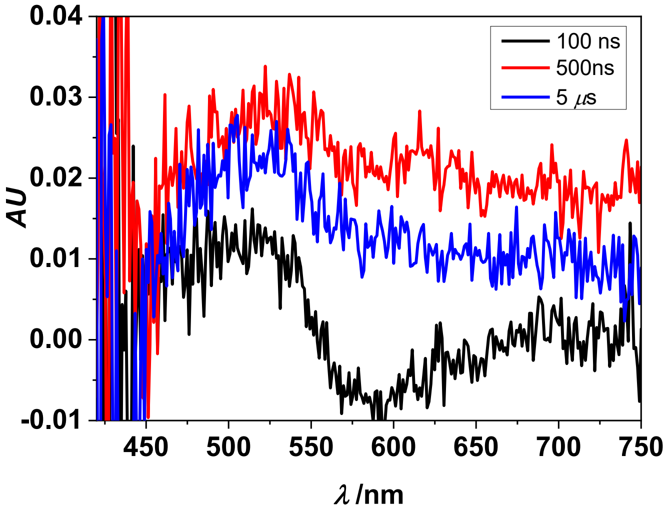

Figure S45. Nanosecond transient absorption spectra of **2** in degassed methanol solution (λ_{ex} = 355 nm).

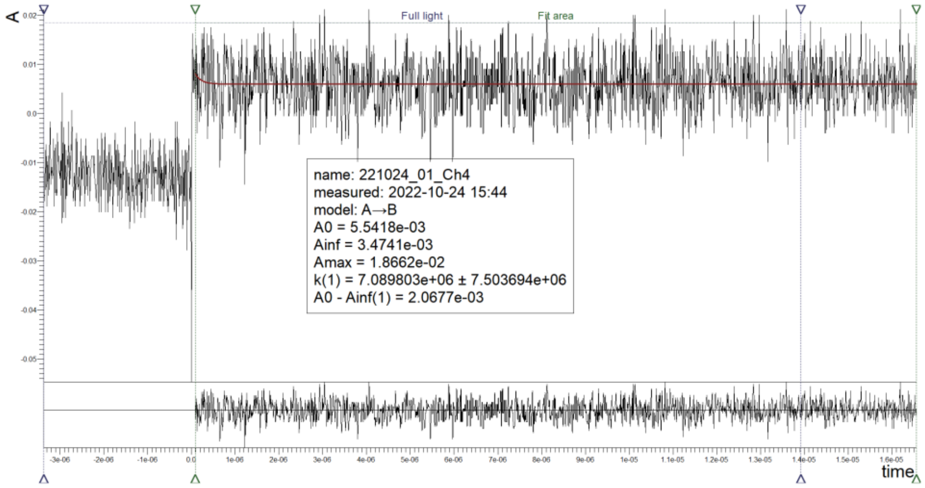

Figure S46. Kinetic decay of a transient formed from **2** in degassed methanol solution (λ_{ex} = 355 nm) and measured at the absorption maximum; the sum of residuals of mono-exponential fitting is at bottom.

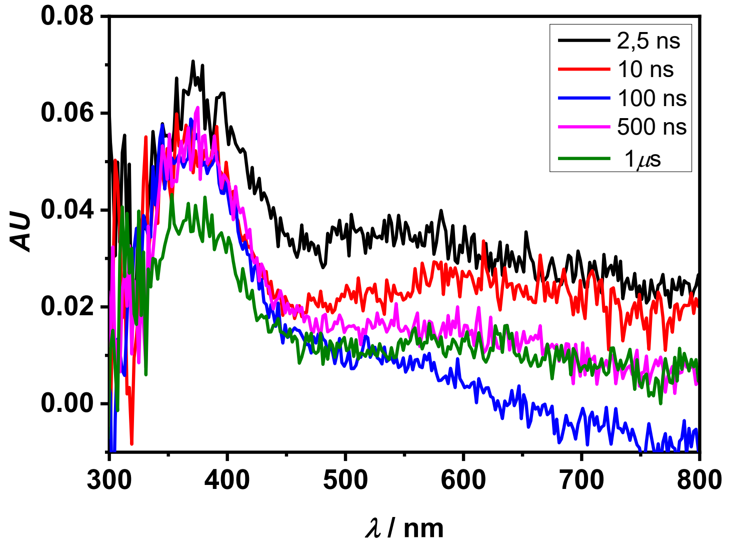

Figure S47. Nanosecond transient absorption spectra of **3** in degassed methanol solution (λ_{ex} = 355 nm).

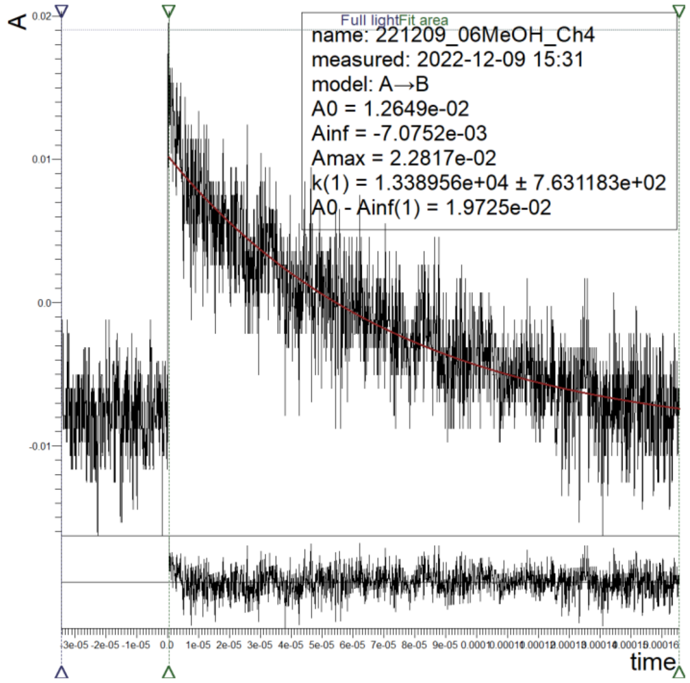

Figure S48. Kinetic decay of a transient formed from **3** in degassed methanol solution (λ_{ex} = 355 nm) and measured at the absorption maximum; the sum of residuals of mono-exponential fitting is at bottom.

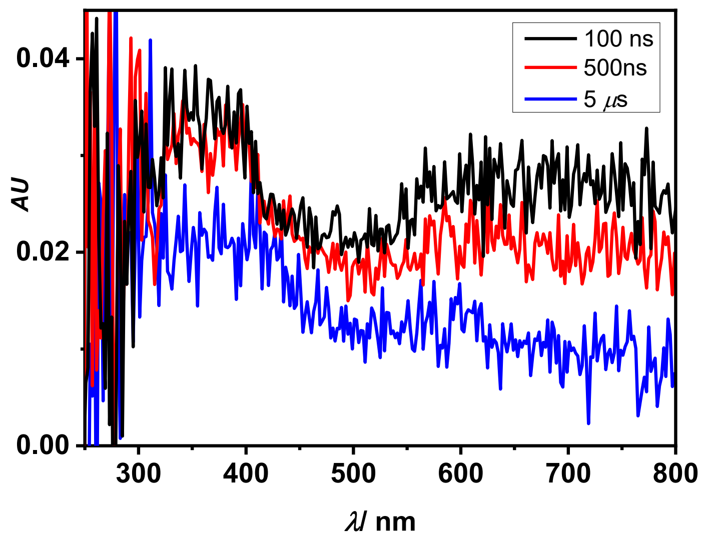

Figure S49. Nanosecond transient absorption spectra of **5** in degassed methanol solution (λ_{ex} = 355 nm).

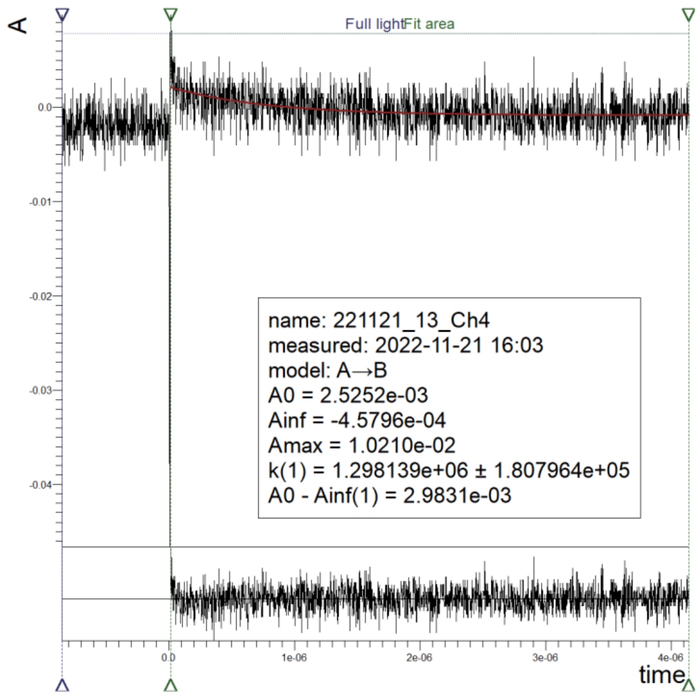

Figure S50. Kinetic decay of a transient formed from **5** in degassed methanol solution (λ_{ex} = 355 nm) and measured at the absorption maximum; the sum of residuals of mono-exponential fitting is at bottom.

9. Emission Spectra of the Irradiation Sources

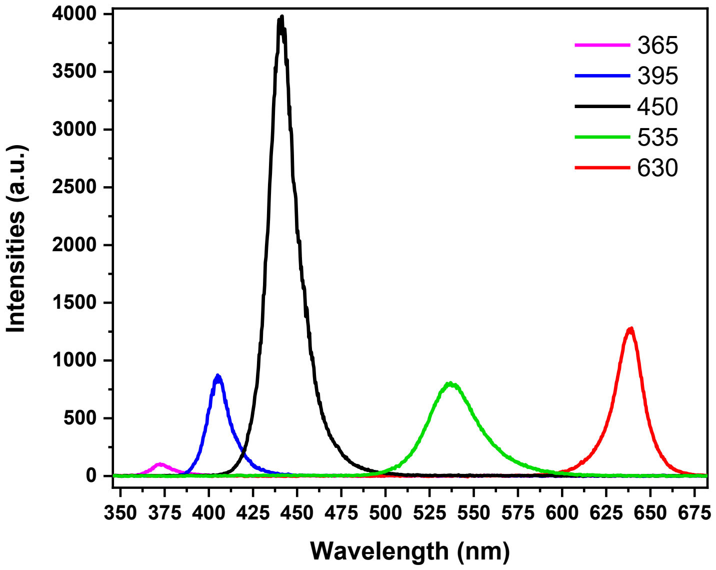

Figure S51. Emission spectra of the LEDs used for irradiations.

10. GC-Headspace Chromatograms.

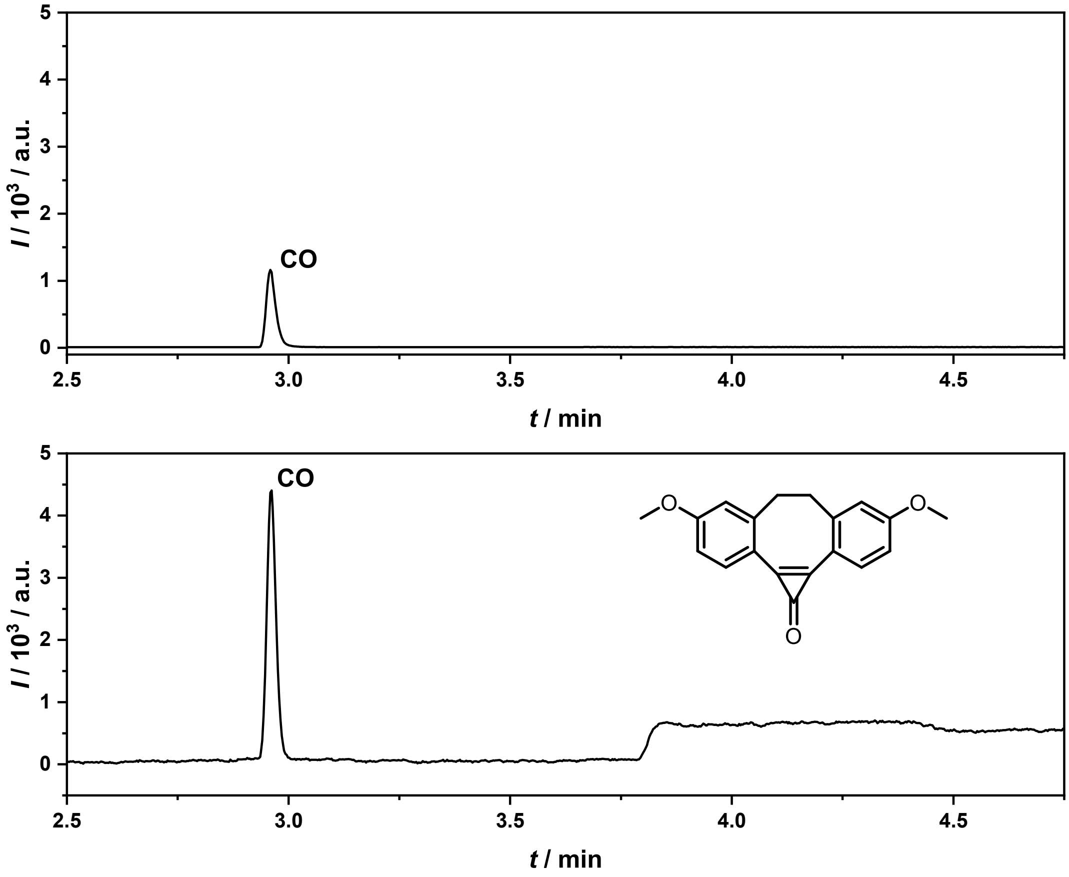

Figure S52. GC-headspace chromatograms. (top) CO produced from **2A** (c ~1 × 10^{-4} M) in methanol upon irradiation at 395 nm for 60 min. (bottom) CO produced from a cyclopropenone photoCORM[1] in methanol upon irradiation with a xenon lamp (cut-off wavelength of a GC-vial was 340 nm) for 30 s.

11. References

1. Martínek, M.; Filipová, L.; Galeta, J.; Ludvíková, L.; Klán, P., Photochemical formation of dibenzosilacyclohept-4-yne for Cu-free click chemistry with azides and 1,2,4,5-tetrazines. *Org. Lett.* **2016,** *18*, 4892-4895.
2. Haag, W. R.; Hoigne, J.; Gassman, E.; Braun, A. M., Singlet oxygen in surface waters — Part I: Furfuryl alcohol as a trapping agent. *Chemosphere* **1984,** *13*, 631-640.
3. Redmond, R. W.; Gamlin, J. N., A compilation of singlet oxygen yields from biologically relevant molecules. *Photochem. Photobiol.* **1999,** *70*, 391-475.
4. Young, R. H.; Brewer, D.; Keller, R. A., Determination of rate constants of reaction and lifetimes of singlet oxygen in solution by a flash photolysis technique. *J. Am. Chem. Soc.* **1973,** *95*, 375-379.
5. Hasse, K.; Willis, A. C.; Banwell, M. G., Modular total syntheses of lamellarin G trimethyl ether and lamellarin S. *Eur. J. Org. Chem.* **2011,** *2011*, 88-99.
6. Sobottka, A. M.; Werner, W.; Blaschke, G.; Kiefer, W.; Nowe, U.; Dannhardt, G.; Schapoval, E. E. S.; Schenkel, E. P.; Scriba, G. K. E., Effect of flavonol derivatives on the carrageenin-induced paw Edema in the rat and inhibition of cyclooxygenase-1 and 5-lipoxygenase in vitro. *Arch. Pharm. Pharm. Med. Chem.* **2000,** *333*, 205-210.
7. Zhang, J.; Liu, M.; Cui, W.; Yang, J.; Yang, B., Total synthesis of luteolin. *J. Chem. Res.* **2014,** *38*, 60-61.
8. Jiang, W.-J.; Ishiuchi, K. i.; Furukawa, M.; Takamiya, T.; Kitanaka, S.; Iijima, H., Stereospecific inhibition of nitric oxide production in macrophage cells by flavanonols: synthesis and the structure–activity relationship. *Bioorgan. Med. Chem.* **2015,** *23*, 6922-6929.
9. Anderson, N. G., Using continuous processes to increase production. *Org. Process Res. Dev.* **2012,** *16*, 852-869.
10. Xiang, Y.; Hu, C.; Zhang, Y.; Ye, X., Synthesis, biological evaluation, and molecular docking of dihydroflavonol derivatives as anti-inflammatory agents. *Med. Chem. Res.* **2019,** *28*, 863-872.
11. Chu, H.-W.; Wu, H.-T.; Lee, Y.-J., Regioselective hydroxylation of 2-hydroxychalcones by dimethyldioxirane towards polymethoxylated flavonoids. *Tetrahedron* **2004,** *60*, 2647-2655.
12. Guo, G.; Wan, S.; Si, X.; Jiang, Q.; Jia, Y.; Yang, L.; Zhou, W., From simple to complex: Rhodium(III)-catalyzed C–C bond cleavage and C–H bond functionalization for the synthesis of 3a,8b-dihydro-1*H*-cyclopenta[b]benzofuran-1-ones. *Org. Lett.* **2017,** *19*, 5026-5029.
13. Xiong, W.; Wang, X.; Shen, X.; Hu, C.; Wang, X.; Wang, F.; Zhang, G.; Wang, C., Synthesis of flavonols via pyrrolidine catalysis: Origins of the selectivity for flavonol versus aurone. *J. Org. Chem.* **2020,** *85*, 13160-13176.
14. Russo, M.; Orel, V.; Stacko, P.; Srankova, M.; Muchova, L.; Vitek, L.; Klan, P., Structure–photoreactivity relationship of 3-hydroxyflavone-based CO-releasing molecules. *J. Org. Chem.* **2022,** *87*, 4750-4763.
15. Anderson, S. N.; Richards, J. M.; Esquer, H. J.; Benninghoff, A. D.; Arif, A. M.; Berreau, L. M., A structurally-tunable 3-hydroxyflavone motif for visible light-induced carbon monoxide-releasing molecules (CORMs). *ChemistryOpen* **2015,** *4*, 590-594.
16. Endo, H.; Nikaido, Y.; Nakadate, M.; Ise, S.; Konno, H., Structure activity relationship study of curcumin analogues toward the amyloid-beta aggregation inhibitor. *Bioorg. Med. Chem. Lett.* **2014,** *24*, 5621-5626.
